# Bluetongue in India: a systematic review and meta-analysis with emphasis on diagnosis and seroprevalence

**DOI:** 10.1080/01652176.2020.1810356

**Published:** 2020-09-23

**Authors:** Ramkumar N. Rupner, O. R. VinodhKumar, R. Karthikeyan, D. K. Sinha, K. P. Singh, Z. B. Dubal, Shikha Tamta, V. K. Gupta, B. R. Singh, Y. S. Malik, K. Dhama

**Affiliations:** a Division of Epidemiology, ICAR-Indian Veterinary Research Institute, Bareilly, India; b Division of Pathology, ICAR-Indian Veterinary Research Institute, Bareilly, India; c Division of Veterinary Public Health, ICAR-Indian Veterinary Research Institute, Bareilly, India; d CADRAD, ICAR-Indian Veterinary Research Institute, Bareilly, India; e Division of Biological Standardisation, ICAR-Indian Veterinary Research Institute, Bareilly, India

**Keywords:** Bluetongue, sheep, goat, cattle, camel, Mithun, diagnosis, seroprevalence, meta-analysis, India

## Abstract

Bluetongue (BT) is an infectious viral disease which affects a wide range of ruminants and was first reported in India in 1964. In view of the absence of comprehensive information on the BT status in India, this study presents the seroprevalence on BT in farm animals of India based-on a systematic review and meta-analysis. A systematic review was conducted to identify the published articles (2001–2018) reporting the seroprevalence of BT in sheep, goats, cattle, buffalo, camels, and Mithun (*Bos frontalis*) from India. From 409 research articles, 71 fulfilled the inclusion criteria and meta-analysis for proportions was carried out targeting the eligible studies. From these, 144 strata level data were extracted with a sample size of 14048 sheep, 14696 goats, 5218 cattle, 2653 buffaloes, 2062 camels, and 222 Mithun. Overall, the analyses showed that the BT seroprevalence of 43% (95% CI: 38–49%) in goats, 39% (95% CI: 33–46%) in sheep, 38% (95% CI: 25–45%) in cattle, 34% (95% CI: 20–51%) in buffaloes, 16% (95% CI: 10–22%) in camels, and 66% (95% CI: 17–95%) in Mithun. Furthermore, the meta-regression analysis suggested that serological tests, geographical region, and sample size were the prime moderators. Meta-analytic study indicates the BT seropositivity in 25.35 million sheep (95% CI: 21.5–29.9), 58 million goats (95% CI: 51.3–66.2), 66.8 million cattle (95% CI: 47.7–86), 37.0 million buffaloes (95% CI: 21.7–55.4), 0.06 million camels (95% CI: 0.04–0.09), and 0.19 million Mithun (95% CI: 0.05–0.28). The findings highlight the variation of BT seropositivity in different geographical regions of India.

## 1. Introduction

Bluetongue (BT) is an insect-borne infectious viral disease of domestic and wild ruminants. It is caused by Bluetongue virus (BTV) and transmitted by *Culicoides* species (Mellor et al. 1984; Rao et al. 2016). The virus belongs to the genus *Orbivirus* of the family *Reoviridae* (Pringle 1999). Sheep are considered as the most susceptible hosts for BT, whereas cattle, buffalo and goats serve as reservoirs. BTV distribution depends on the vector presence, and it is endemic in geographical regions located in latitude of approximately 40°–50° North and 35° South (Walton 2004). Nowadays, the BTV distribution range is extending to temperate zones due to climate change (Purse et al. 2005). In India, BT was first reported from Maharashtra state in 1964 (Sapre 1964). Since then, reported from several states and union territories of the country. Now, BT is endemic in India and serological evidence exists from several states of India in sheep, goats, cattle, buffaloes, camels, Mithun and also some captive wild ruminants.

To date, 27 distinct BTV serotypes have been described, of which 24 typical serotypes and other atypical novel BTV serotypes including BTV-25 (Toggenburg virus strain), BTV-26, BTV-27 (variants 01, 02 and 03), BTV-29, BTV-XJ1407, and BTV-X ITL2015 have been identified worldwide (Wright 2014; Zientara et al. 2014; Schulz et al. 2016; Sun et al. 2016; Savini et al. 2017; Marcacci et al. 2018; Saminathan et al. 2020). Of note, a novel BTV serotype was detected in a batch of commercially contaminated sheep pox vaccine (Rajko-Nenow et al. 2020). In India, at least 22 serotypes have been recognized based on serology and/or virus isolation. As of now, 13 serotypes viz., BTV-1, 2, 3,4, 6, 9, 10, 12 16, 17, 18, 21, and 23 have been isolated within the All India Network Program on Bluetongue (AINP-BT) and other research laboratories (AINP-BT 2012; Rao et al. 2016).

The diagnosis of BTV infection is based on either pathogen identification or immune response detection. Real-time RT-PCR, RT-PCR (Reverse transcriptase polymerase chain reaction), and classical virus isolation are the methods for pathogen identification (Lakshmi et al. 2018). For the detection of the immune response in the host, c-ELISA (Competitive enzyme linked immunosorbent assay) (serogroup specific), virus neutralization test (VNT, serotype-specific), agar gel immunodiffusion test (AGID), and complement fixation test (CFT) are available . In earlier times, CFT was used to detect BTV antibodies and now it is replaced by the AGID in many parts of the world. The AGID test is easy and simple to perform but the major disadvantage is that it cannot differentiate between antibodies to the BT and epizootic haemorrhagic disease (EHD) serogroups, thereby enhancing its poor specificity. VNT is used to identify BT serotype-specific neutralizing antibodies (OIE 2014). The OIE recommended serodiagnosis tests are complement fixation test, agar gel immunodiffusion (AGID), competitive enzyme-linked immunosorbent assay (ELISA) and indirect ELISA. Many advanced techniques for detection of both virus and antibody have been developed in different countries. The techniques for virus characterization are Dot immunoperoxidase assay (Clavijo et al. 2000), immunomicroscopy (Nunamaker et al. 1992), viral genome detection by using Southern, Northern blot techniques or dot blots, *in situ* hybridizations and sequence analysis. Regarding antibody detection many serogroup specific assays have been developed along the years and it includes techniques like immunochromatographic strips (ICS) (Yang et al. 2010), protein chip detection (Xu et al. 2016), latex agglutination test (Yang et al. 2010), and double-antigen microsphere immunoassay (MIA) (Breard et al. 2017). Indirect ELISA based on detection of BTV NS3 antibodies can be used for Differentiation of Infected from Vaccinated Animals (DIVA) (Barros et al. 2009). Though there are many techniques developed for BTV diagnosis, the efficacy, time consuming and high cost are major constraints in wide usage of it. In India, most of the seroepidemiological studies are conducted by detecting BTV group-specific antibodies with ELISA and AGID assay (Rao et al. 2016). After the inception of the AINP-BT, the prevalence and distribution of anti-BTV antibodies were carried out as per the OIE recommended monoclonal antibody-based competitive-ELISA (c-ELISA) (OIE 2014).

Severe economic loss due to trade restrictions in outbreaks prone areas makes BTV as notifiable to the World Organization for Animal Health (OIE). Bluetongue causes both direct (production) and indirect (revenue loss) economic impact on livestock. Globally, the estimate of economic loss due to BT was US$3 billion (Rushton and Lyons 2015). In India, the highest annual average loss occurred due to BT was around Rs. 299.09 lakh which accounts for 61% of total economic loss due to all diseases in sheep (Singh and Prasad 2009).

The live attenuated and inactivated BT vaccines showed varying degree of success in BT control. However, the modern vaccine such as subunit recombinant vector BT vaccines evidenced better protective immune response (Ranjan et al. 2019). Control and eradication of BT is hardly possible due to the presence of asymptomatic infections, prolonged viremia in cattle and virus persistence in the vector population maintains virus in the environment. Moreover, the movement restrictions and stamping out of the diseased animals are very difficult. Better diagnostic methods, surveillance system and vaccination with circulating serotypes are possible methods to reduce and control the BT infection (OIE 2014). Recent reports suggest that composition of livestock is the influencing factor for spatial patterns of bluetongue in Southern India (Chanda et al. 2019).

As reports on BT are available from different states and different time-periods, the unified data on the status of BT in India is lacking. To abridge this critical knowledge gap, a systematic review and meta-analysis was performed to determine the BT seroprevalence in domestic animals of India and also to provide valuable inputs in formulating the disease control strategies.

## 2. Materials and methods

### 2.1. Literature search strategy

For this systematic review and meta-analysis, a literature search was performed to identify all peer-reviewed articles reporting seroprevalence of bluetongue in India. A preliminary article search was conducted using ScienceDirect, Scopus, Indianjournals.com, PubMed, J-Gate @Consortium of e-Resources in Agriculture (CeRA) under Indian Council of Agricultural Research, Google scholar, Springer and handpicked publications (2001–2018). The keywords used for the search included the boolean search strings consisted of bluetongue, sheep, goat, Mithun, cattle, buffalo, small ruminants, domestic animals, India, epidemiology, risk factors, prevalence, seroprevalence and serotype.

### 2.2. Eligibility conditions and data extraction procedure

**Figure 1.**
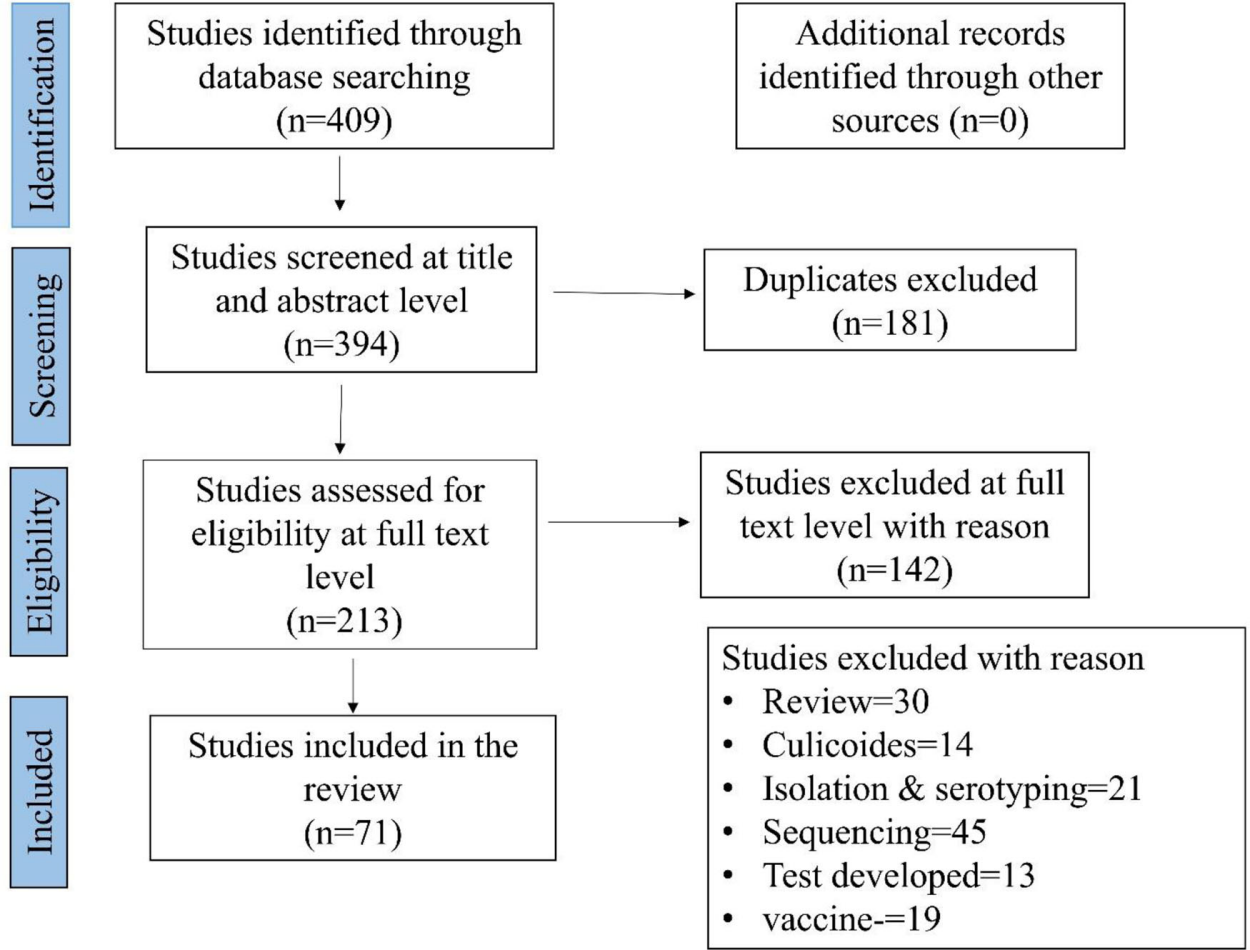
Schematic diagram showing the studies from India on BT seroprevalence (2001–2018) included for meta-analysis.

All the articles that reported BT in India were collected for the analysis. Quality criteria were developed using Meta-analysis of Observation Studies in Epidemiology (MOOSE), and Preferred Reporting Items for Systematic Reviews (PRISMA) protocol (Shamseer et al. 2015). The title, abstract, full-text screening, data extraction, and quality assessment were also carried out before starting the review of full papers. All these studies were reviewed and screened manually by two investigators independently using both inclusion and exclusion criteria which were set depending upon the objective of the study and the disagreement between the two investigators were resolved by the third investigator. The schematic representation of the literature selection procedure for the systematic review of BT seroprevalence in India is depicted in Figure 1. The relevant papers were retained, and the results extracted from papers include the author's name, article title, year of publication, sample size, number of positives, study area, study year and diagnosis method used. From the 409 publications screened (from 2001 to 2018), 71 articles were incorporated in the systematic review and meta-analysis. The proportions for BT seroprevalence in domestic animals were carried out using 71 studies with 144 strata level data extracted from these studies, which resulted in a total sample size 38,899 (14048 sheep, 14696 goats, 5218 cattle, 2653 buffaloes, 2062 camels, and 222 Mithun). To observe the effect of time on the BT seroprevalence, the study periods were classified into four-time periods such that each period having four to five years duration: 2001–05; 2006–09; 2010–13; and 2014–2018. Furthermore, to observe the influence of sample size on the BT seroprevalence, the sample size was classified into three categories based on the sample sizes of the included studies and to have sufficient number of studies in each category: <200; 201–500 and >500 samples. The states which reported the seroprevalence of BT were categorized into following six regions- (i) Northern region - Jammu and Kashmir, Punjab, Uttar Pradesh, Uttarakhand; (ii) Eastern region - West Bengal, Odisha, Bihar, Jharkhand; (iii) Northeast Region- Assam, Tripura, Meghalaya, Nagaland; (iv) Western region- Rajasthan, Gujarat, Maharashtra, (v) Central region- Madhya Pradesh, Chhattisgarh; and (vi) Southern region - undivided Andhra Pradesh, Karnataka, Kerala, and Tamil Nadu. The included studies used serodiagnostic techniques AGID, ELISA, c-ELISA, sandwich-ELISA (sELISA), DOT-ELISA, and Counter immunoelectrophoresis (CCIE). Some studies performed multiple tests; for example, the study by Chandel et al. (2004), Chauhan et al. (2004), and Patel et al. (2017). The meta-analysis description of the included studies is shown in Table 1. The details of included and excluded studies are given in supplementary files.

**Table 1.** Details of the sub-group analysis for seroprevalence of bluetongue in India.

S. No	Variables		Studies included	Strata level Studies	Samples tested	Positive samples	Pooled estimate (RE) (95% CI)	Pooled estimate (FE) (95% CI)	Q Test	*p* value	I^2^ value	Tau square
1.	Animals	Goat #	44	44	14696	6294	43% (38%-49%)	45% (44%−46%)	1739.55	0.00	98%	0.59
		Sheep #	40	47	14048*	5862	39% (33%−46%)	41% (40%−41%)	1618.26	0.00	98%	0.05
		Cattle #	21	28	5218	2149	35% (25%−45%)	40% (39%−41%)	1494.63	<0.01	98%	0.07
		Buffalo#	14	16	2653**	1134	34% (20%−51%)	42% (41%−44%)	458.09	<0.01	98%	0.11
		Camel	07	07	2062	449	16% (10%−22%)	21% (19%−23%)	73.36	<0.01	92%	0.01
		Mithun	02	02	222	167	66% (17%−95%)	73% (65%−79%)	39.98	<0.01	97%	2.60
2.	Geographic region	North zone	11	17	3773	1753	41% (27%−56%)	47% (45%−48%)	867.48	<0.01	98%	1.52
		West zone	28	60	14668	5763	37% (32%−41%)	40% (39%−41%)	1435.93	<0.01	96%	0.47
		Central zone	05	13	5776	2673	46% (39%−54%)	46% (45%−48%)	378.77	<0.01	97%	0.30
		South zone	14	28	6173	2011	28% (18%−41%)	40% (38%−42%)	1554.52	<0.01	98%	2.02
		East zone	09	15	5109	2173	46% (37%−55%)	42% (40%−43%)	514.66	<0.01	97%	0.54
		North East Zone	08	11	2868	1414	44% (34%−54%]	50% (48%−52%)	218.68	<0.01	95%	0.46
3.	Serological test	I-ELISA^	19	31	9792	4113	40% (35%−45%)	43% (42%−44%)	592.22	<0.01	95%	0.27
		AGID^	14	28	6345	1254	19% (14%−24%)	18% (17%−19%)	727.25	<0.01	96%	0.03
		C-ELISA^	35	70	20746	10341	52% (46%−57%)	50% (49%−51%)	3896.32	<0.01	98%	0.05
		S-ELISA^	04	08	514	155	25% (14%−41%)	40% (35%−45%)	53.58	<0.01	87%	0.74
		ELISA	02	03	141	44	25% (5%−68%)	37% (28%−48%)	34.49	<0.01	94%	2.66
		DOT-ELISA	01	02	1010	57	6% (4%−9%)	6% (4%−7%)	1.54	0.21	35%	0.04
		CCIE	02	02.	351	91	24% (6%−58%)	24% (22%−27%)	31.56	<0.01	97%	1.16
4.	Study period	2001-05	17	41	8808	2965	28 % (22%−35%)	36% (35%−37%)	1330.02	<0.01	97.%	0.89
		2006-09	15	28	7710	3323	40% (32%–49%)	48% (47%−49%)	1183.01	<0.01	97%	0.88
		2010-13	16	32	8431	3660	43% (37%–50%)	44% (42%−45%)	905.71	<0.01	96%	0.52
		2014-18	23	43	13418	5839	41% (35%–47%)	43% (42%−44%)	1515.38	<0.01	97%	0.56
5.	Sample size	<200 ^@^	42	84	8001	3038	33% (29%–38%)	41% (40%−42%)	1302.03	<0.01	94%	0.84
		201-500 ^@^	21	36	11347	5790	51% (43%–58%)	50% (49%−51%)	1899.34	<0.01	98%	0.87
		>500 ^@^	21	24	19019	6959	32% (26%–38%)	39% (39%−40%)	1629.37	<0.01	98%	0.43

* includes the study of Shringi and Shringi (2005) in which the same samples (n = 178) were tested by both CCIE and c-ELISA; ** includes the study of Patel et al. (2007) in which the same samples (n = 173) were tested by both CCIE and c-ELISA. # includes studies on seroprevalence in more than one species of animals. ^ includes studies with more than one serodiagnostic test used on animals. @ includes studies with different sample sizes.

### 2.3. Statistical analysis

Summary reports on BT seroprevalence was performed by using descriptive statistics. Between-study heterogeneity was assessed graphically by visual inspection of Baujat plot and quantified by Higgin's I^2^ and Cochran's Q method. If the heterogeneity test had a *p*-value < 0.1, a random-effects (RE) model was used. Otherwise, the fixed-effects (FE) model was used. The meta-analysis on BT seroprevalence was performed through random effect model using inverse-variance model (DerSimonian and Laird 1986). Freeman Tukey double arcsine transformation was used for pooling seroprevalence from raw cell counts (Harris et al. 2008; Nyaga et al. 2014). The pooled estimate was measured and reported as seroprevalence, with point and, 95% confidence intervals (CI) and prediction intervals (PI). Forest plots were used to visualize the seroprevalence in each study and the combined estimated seroprevalence. Publication bias was assessed graphically by visual inspection of the funnel plot, and Egger method was used if more than 10 studies are included in the analysis (Egger et al. 1997; Deeks et al. 2008). A set of case deletion diagnostics such as studentized residuals, difference in fits values (DFFITS), Cook’s distances, COVRATIO and leave-one-out estimates, for the amount of heterogeneity as well as the test statistic for heterogeneity were used to identify the influential studies (Viechtbauer and Cheung 2010). The sensitivity analysis was carried out with and without the exclusion of influential studies to verify the robustness of the study design, sample size, study conclusions and the effect of missing data.

Subgroup analysis was conducted to identify the stratified seroprevalence in different regions, study period, sample size, diagnostic tests and species. To predict the effect of a hypothesized moderator, a weighted linear regression model was applied in which the effect sizes (double-arcsine transformed proportions) were regressed onto the moderator (Higgins and Thompson 2002; Card 2015). The moderators included in univariate meta-regression were serological test, study period, geographic region, diagnostic tests, and categorized sample size. The variables with *p* < 0.1 in univariate meta-regression were used in multivariable meta-regression, and only factors significant at *p* ≤ 0.05 were retained in the final model. The statistical analyses were conducted using the R statistical platform (R Foundation for Statistical Computing, Vienna, Austria version 3.5.1) with ‘meta’ package (version 4.9-2) and ‘metafor’ package (version 2.0-0).

## 3. Results

### 3.1. Information on included studies

From the 409 publications screened (from 2001 to 2018), 71 articles were incorporated in the systematic review (Figure 1) and meta-analysis. The proportions for BT seroprevalence in domestic animals were calculated using 71 studies with 144 strata level data extracted from these studies. For example, the study by Bhanuprakash et al. (2008) was extracted into four strata level representing different states where the study was performed, while the study by Ayanur et al. (2016) was extracted into four strata level data, the strata being the two states and two species. The same procedure was repeated for other studies that included data on different study year, location, diagnostic test, species, etc. The studies included in the quantitative analyses provided BT seroprevalence data for a total sample size of 38,899 (14048 in sheep, 14696 in goats, 5218 in cattle, 2653 in buffaloes, 2062 in camels, and 222 in Mithun).

### 3.2. Meta-analysis

A meta-analysis of these studies showed a significant heterogeneity (Q = 8869.91, I^2^ = 98%, df = 143, *p* < 0.01) between the studies and the between-study variance (Tau square) was 0.06. The pooled seroprevalence for BT in domestic animals using a RE model was 38% (95% CI: 35–43%). The RE model revealed better symmetry than the FE model and indicated that the RE model is a better one. In sub-group analysis, a significant heterogeneity (I^2^ indices >90%, *p* values <0.01) was noticed for all subgroups. In forest plot, the effect estimate and weight of each study were represented by a square, and the whiskers on both sides of the block represented a 95% CI. The midpoint of the box represents the point effect estimate for each study. The dark (red color) line in the forest plot symbolizes the prediction interval (PI) at 95% level that specifies the future prediction of the BT seroprevalence lie within the range.

### 3.3. Publication bias

**Figure 2.**
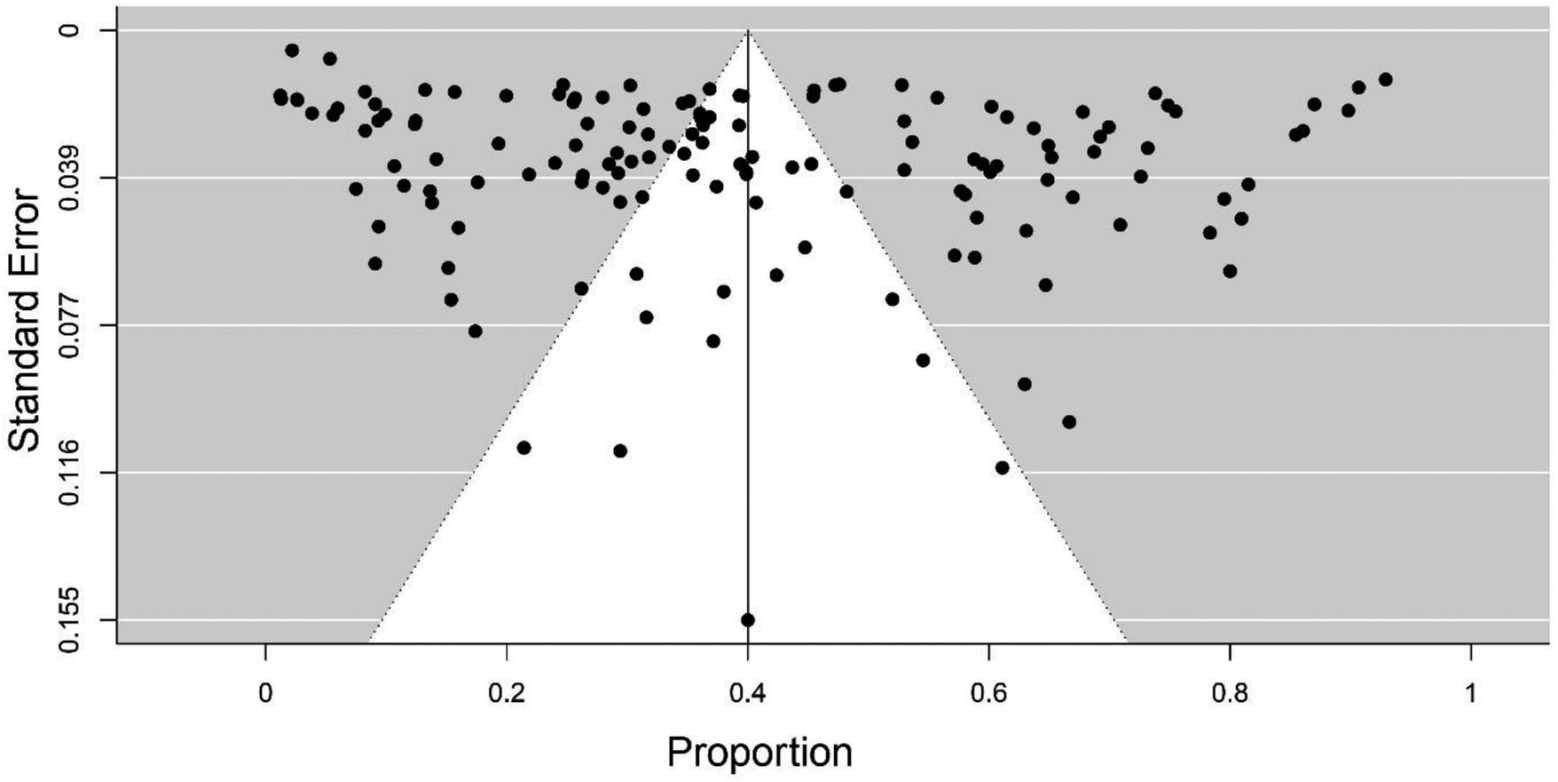
Funnel plot of standard error and seroprevalence demonstrates potential publication bias.

Publication bias was measured using a funnel plot, in which the standard error on Y-axis and proportion of each study on X-axis were plotted, most of the studies were scattered, and a few of the studies fallen into the funnel specifying publication bias (Figure 2). The rank correlation test failed to identify a significant relationship between sample and effect size (Kendall’s tau = 0.007, *p* > 0.05). Regression test (Eggers test) (Z = −2.01, *p* < 0.05) showed that the funnel plot is symmetrical indicating significant publication bias (Figure 2). Owing to significant publication bias the RE model results were considered.

### 3.4. Bluetongue seroprevalence

**Figure 3.**
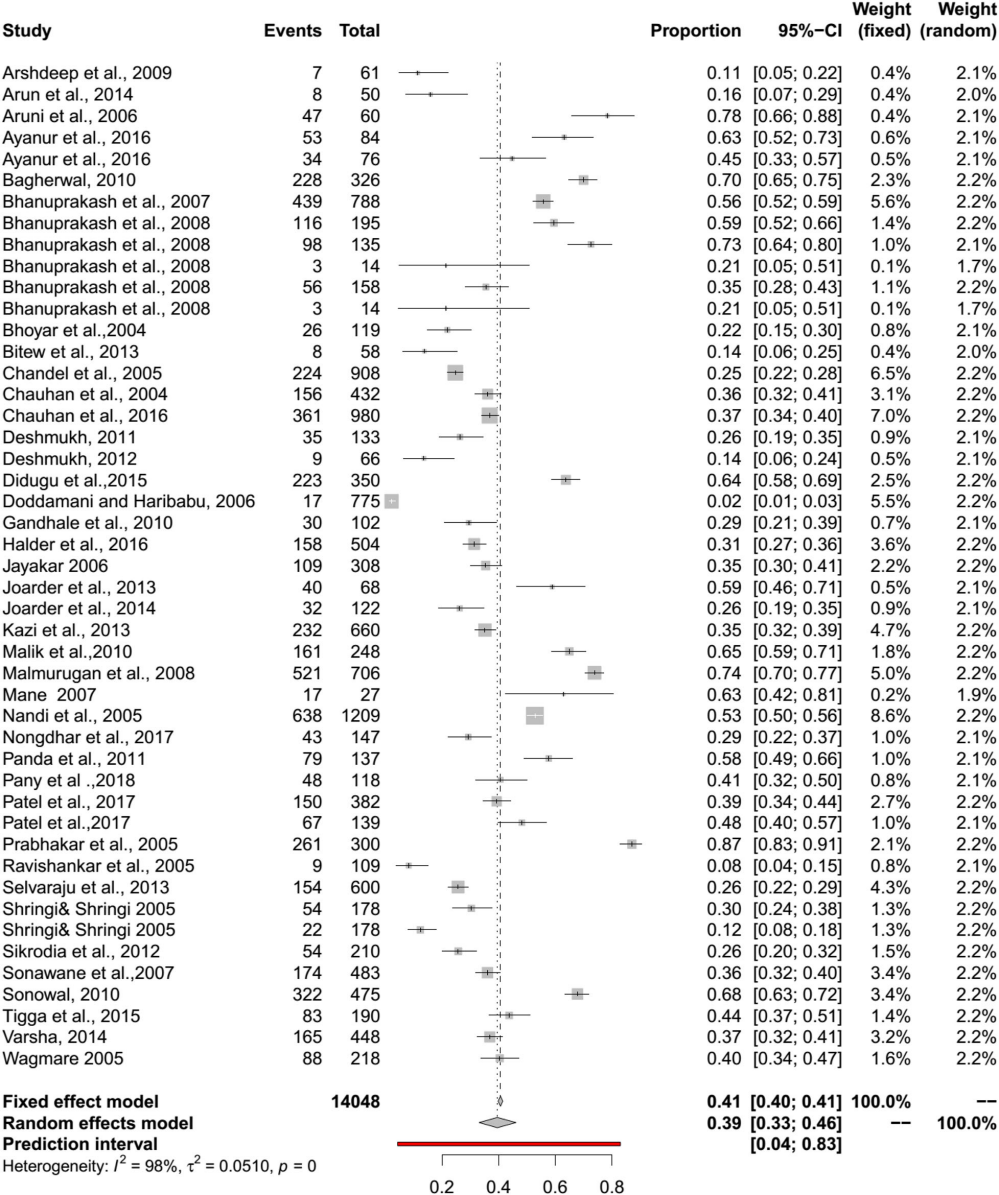
Forest plot visualizing the BT seroprevalence in sheep reported for each included publication in the meta-analysis. Weightage given to each included publication by both RE and FE models have been shown for comparison. ‘Total’ refers to the number of animals in each publication, ‘Events’ refers to the number of BT seropositive animals and ‘Proportion’ refers the BT seroprevalence for each publication.

**Figure 4.**
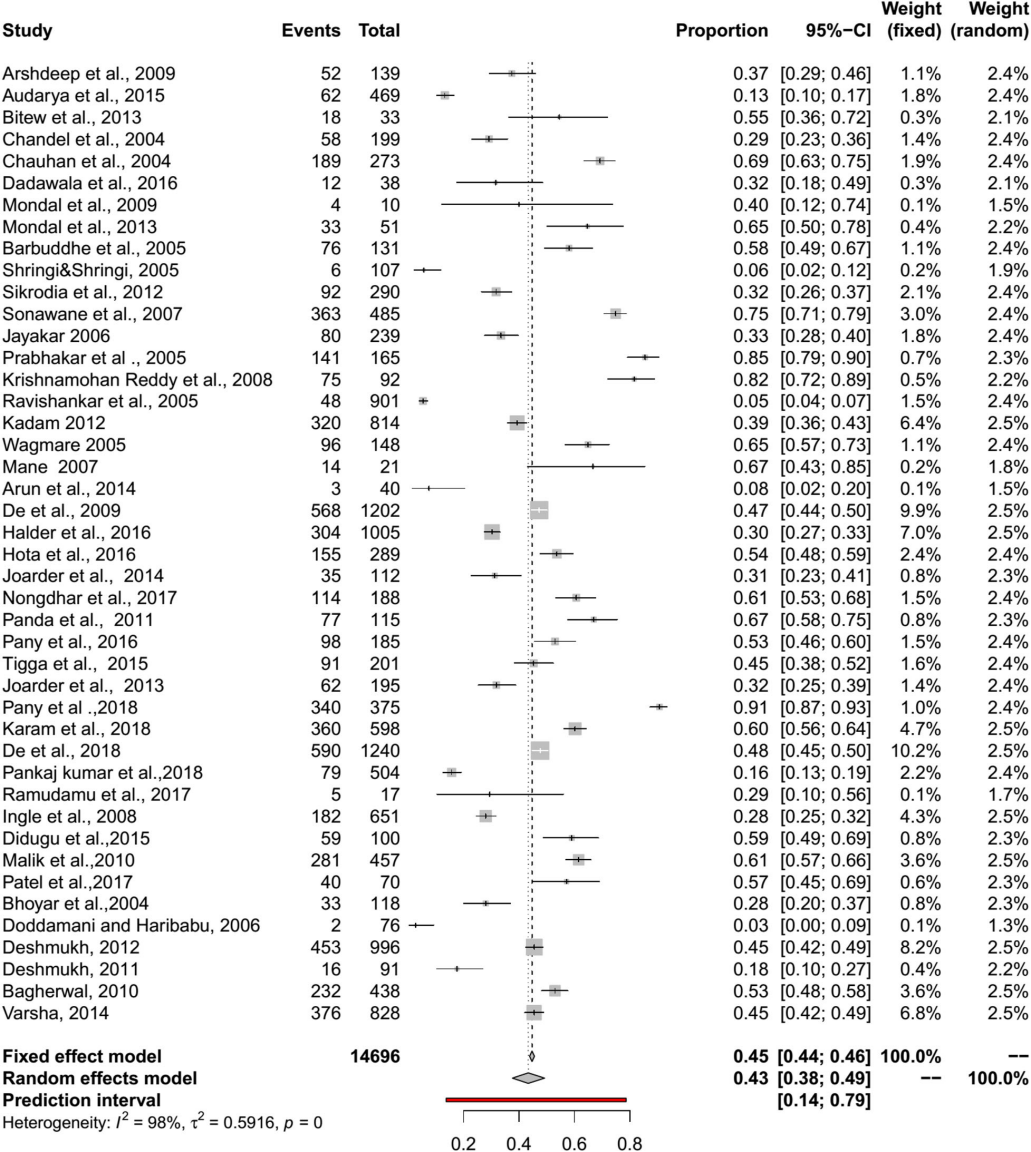
Forest plot visualizing the BT seroprevalence in goat reported for each included publication in the meta-analysis.

The pooled seroprevalence for BT in sheep by RE model was 39% (95% CI: 33–46%; 95% PI: 4–83%; Q = 1618.26, df = 45, *p* < 0.01; tau square = 0.05; I^2^ = 98%) and in goats 43% (95% CI: 38–49%, PI: 14–79%; Q = 1739.55, df = 43, *p* < 0.01, tau square = 0.59, I^2^ = 98%) (Figures 3 and 4). The diagnostic tests sub-group analysis showed that the BT seroprevalence in sheep by c-ELISA was 46% (95% CI: 39–54%) followed by i-ELISA 39% (95% CI: 29–49%), and s-ELISA 39% (95% CI: 21–58%). In goats, seroprevalence by c-ELISA was 55% (95% CI: 46%-65%) followed by i-ELISA 44% (95% CI: 38–50%) and s-ELISA 44% (95% CI: 26–63%).

**Figure 5.**
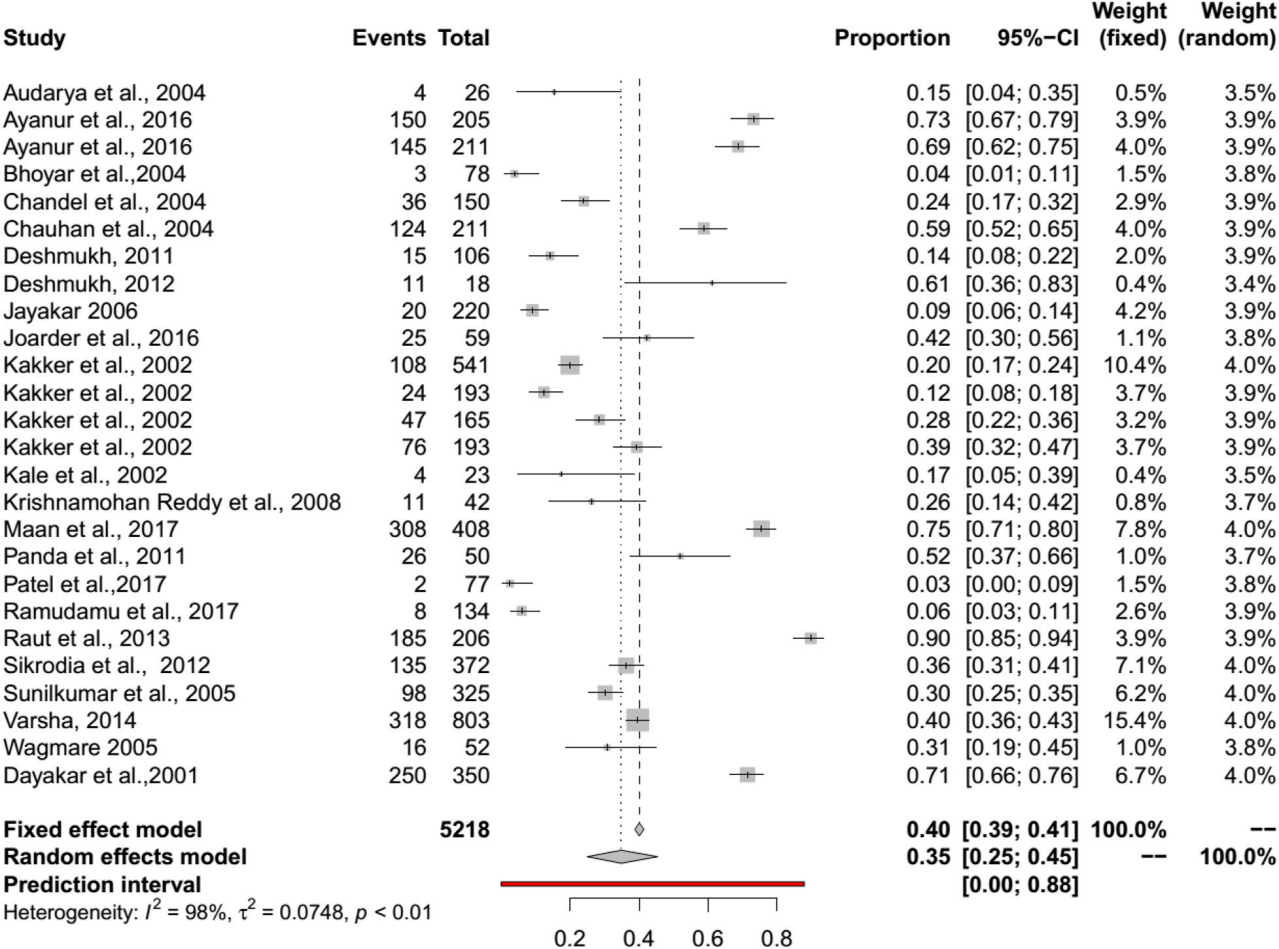
Forest plot visualizing the BT seroprevalence in cattle reported for each included publication in the meta-analysis.

**Figure 6.**
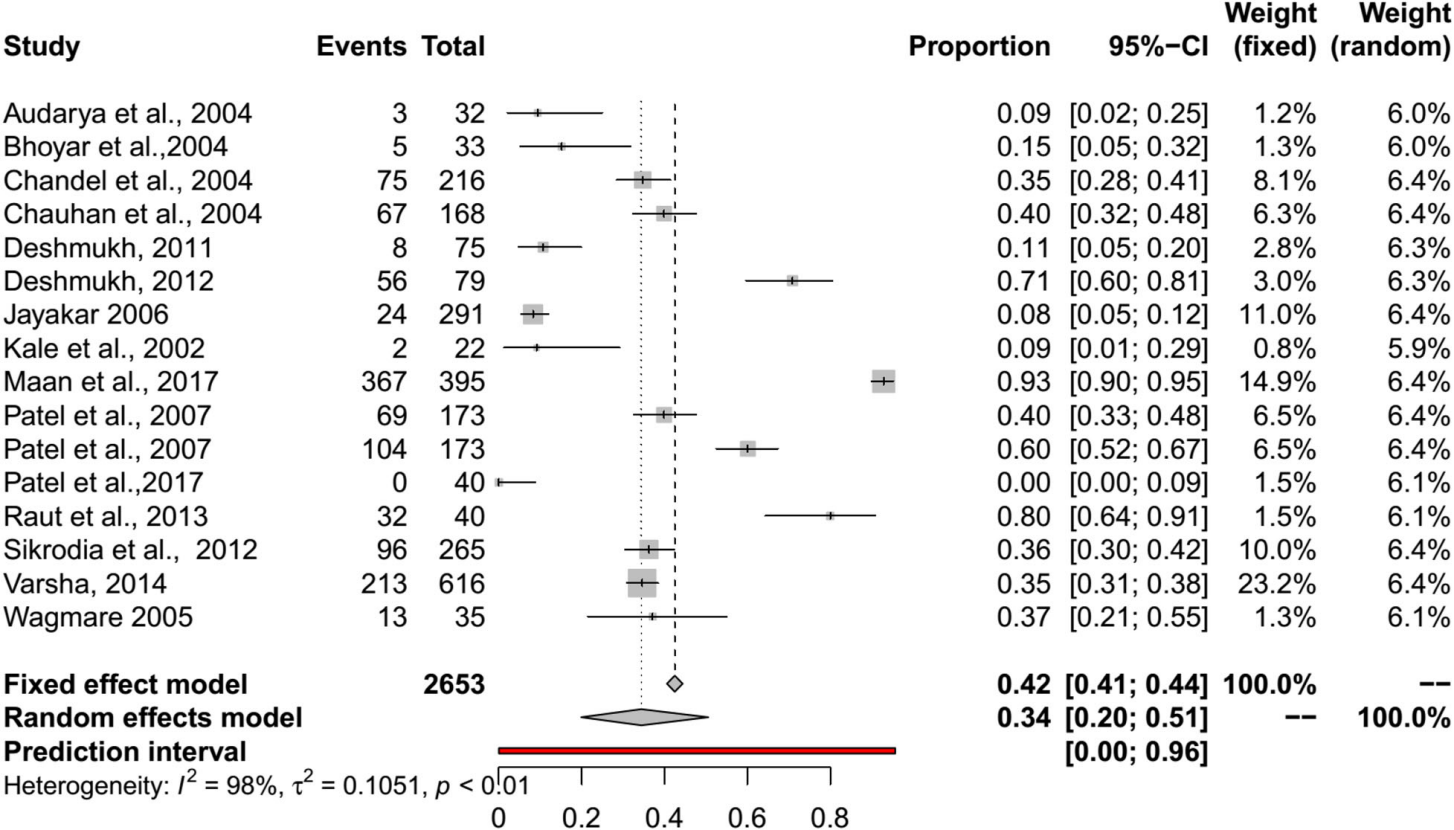
Forest plot visualizing the BT seroprevalence in buffalo reported for each included publication in the meta-analysis.

**Figure 7.**
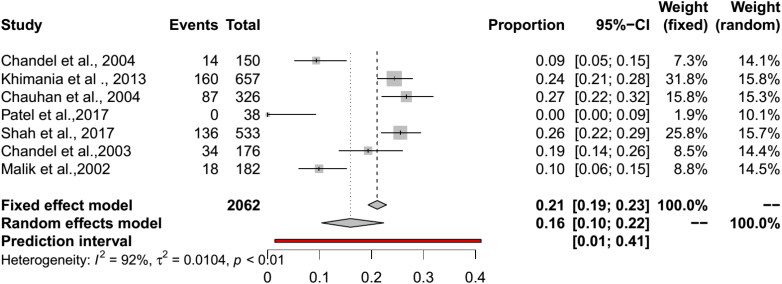
Forest plot visualizing the BT seroprevalence in camel reported for each included publication in the meta-analysis.

**Figure 8.**
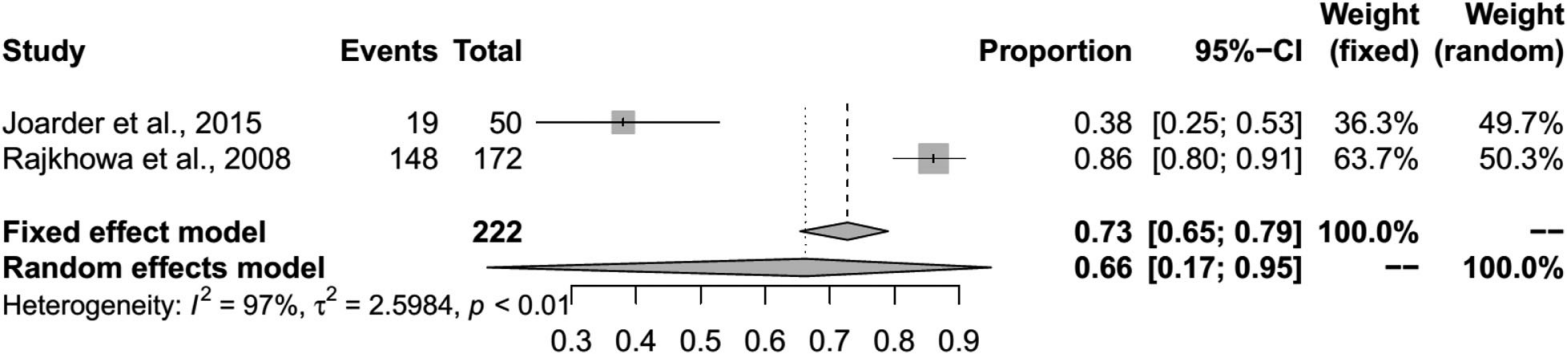
Forest plot visualizing the BT seroprevalence in mithun reported for each included publication in the meta-analysis.

The pooled BT seroprevalence for BT in cattle using the RE model was 35% (95% CI: 25–45%, PI: 0–88%; Q = 1483.28, df = 25, *p* < 0.01, tau square = 0.07, I^2^ = 98%) and in buffaloes it was 34% (95% CI: 20–51%, PI: 0–96%, Q = 458.09, df = 14, *p* < 0.01, tau square = 0.11, I^2^ = 98%) (Figures 5 and 6). In cattle and buffalo, the highest seropositivity was detected by c-ELISA (cattle: 56%, 95% CI: 42–70%; buffalo: 54%, 95% CI: 30–77%) followed by i-ELISA (cattle: 32%, 95% CI: 13–55%; buffalo: 36%, 95% CI: 31–42%) and AGID (cattle: 19%, 95% CI: 13–26%; buffalo: 15%, 95% CI: 4–30%). The seroprevalence for BT in camels by RE model was 16% (95% CI: 10–22%, PI: 1–41%; Q = 73.36, df = 6, *p* < 0.01, tau square = 0.01, I^2^ = 92%) and in Mithun it was 66% (95% CI: 17–95%, Q = 39.98, df = 1, *p* < 0.01, tau square = 2.60, I^2^ = 97%) (Figures 5–8). The analysis showed cELISA as a better diagnostic test for BT seroprevalence.

**Figure 9.**
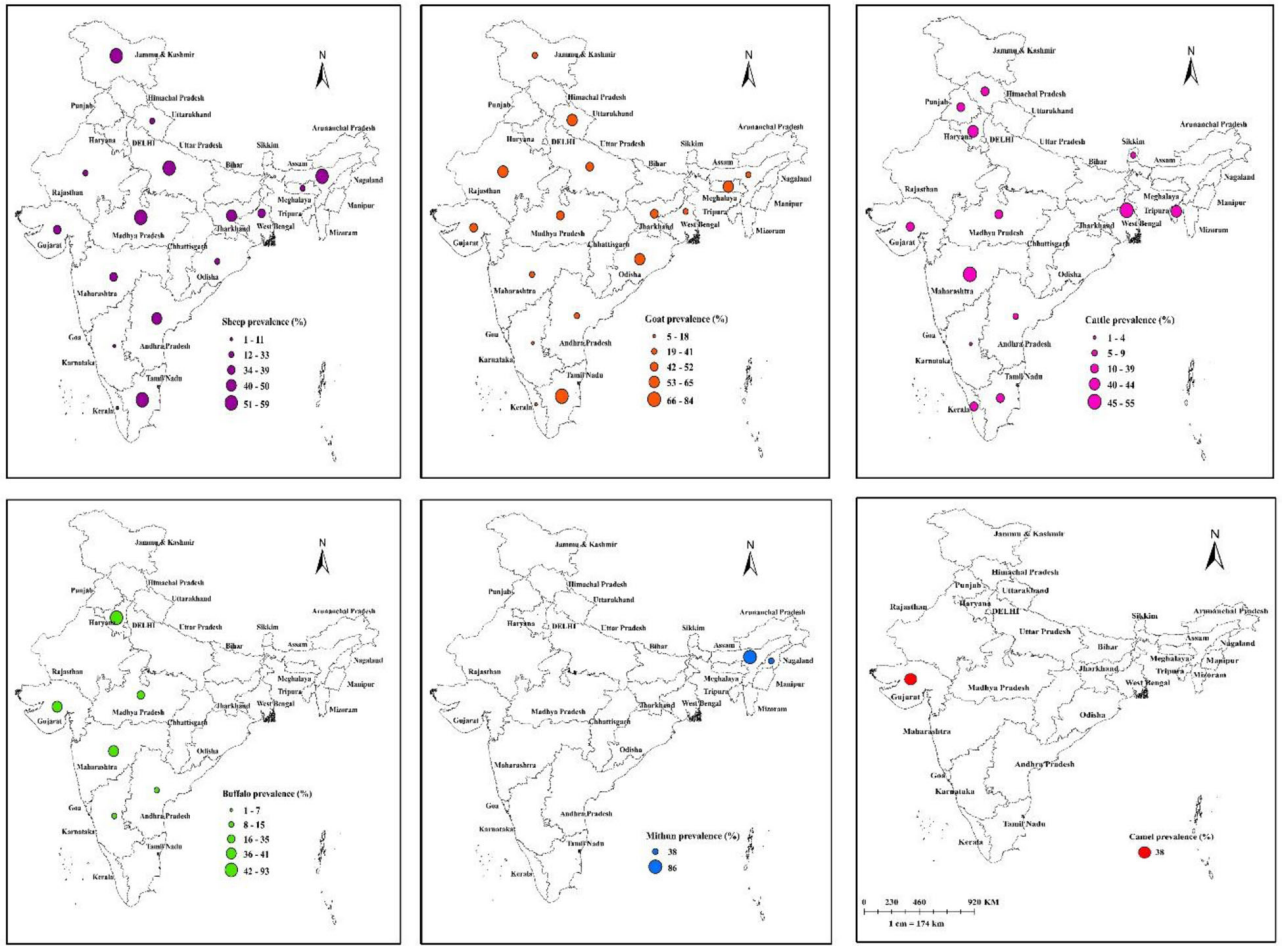
Proportional circle map depicting the species wise seroprevalence of BT in India.

**Figure 10.**
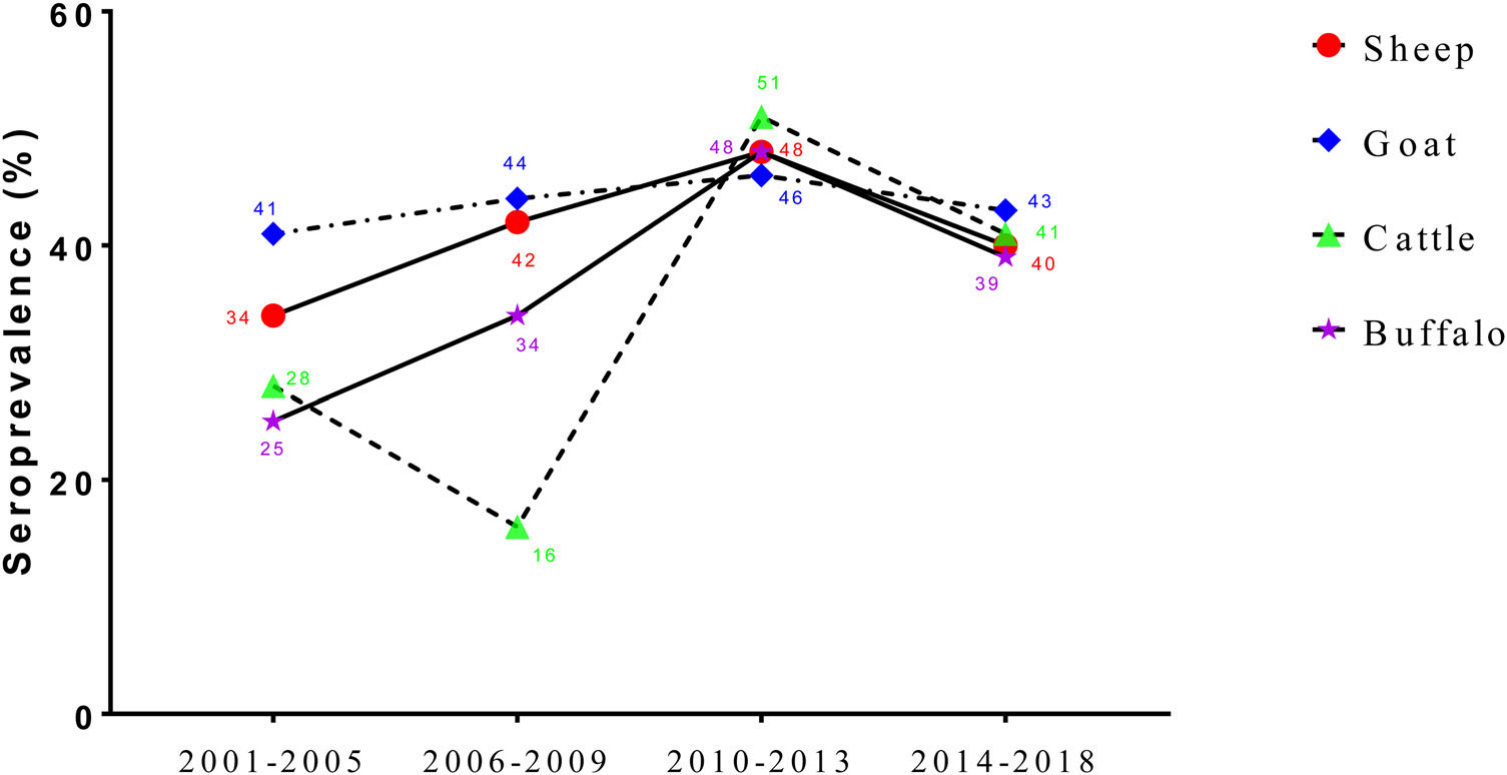
Temporal pattern in the seroprevalence of BT during 2001–2018.

The region wise sub-group analysis showed that the highest BT seroprevalence was noted in sheep from central zone (53%), goats from East zone (48%), cattle from East zone (52%), and buffalo from North zone (93%) (Figure 9). The subgroup analysis with time period showed that the BT seroprevalence in sheep, goats and buffaloes showed an increasing pattern from 2001 to 2013. However, cattle BT seroprevalence showed a significant declining trend from 2001 to 2009. Similarly, declining pattern in the BT seroprevalence was seen in sheep, goats, cattle, and buffaloes from 2010 to 2013 (Figures 10). The diagnostic test, geographic region and study period wise seroprevalence in sheep, goats, cattle and buffaloes are given in supplementary figures.

### 3.5. Heterogeneity, influential study and sensitivity analysis

Baujat plot was used to detect the studies contributing to the overall heterogeneity in the meta-analysis. The studies contributed for overall heterogeneity were two in sheep (study nos. 20 and 37), five in goats (study nos. 10, 14, 16, 30, 40), and one each in cattle (study no 21) and buffaloes (study no 9). Among the studies which contributed to overall heterogeneity, one study each in sheep (study no 37, Doddamani and HariBabu 2006) and in buffaloes (study no 9, Maan et al. 2017) was identified as an influential study. The sensitivity analysis after removing the influential studies revealed that the seroprevalence was marginally increased in sheep from 44% to 46% while decreased from 34% to 31% in buffaloes. No influential studies were noticed for goat, cattle and camel.

### 3.6. Meta-regression analysis

The univariate meta-regression was performed to ascertain the effect of study-level covariates on the pooled BT seroprevalence estimate (Table 2). The heterogeneity contribution by the predictor variables ranged from 0 to 29% in the RE model, and the highest value of R^2^ was observed for serological test while sample size exhibited no effect on heterogeneity (R^2^ = 0%). In multivariate model, the significant predictors for BT seroprevalence were serological tests, geographical region (East and Northeast zone) and sample size (<200 and >500). Analysis of variance (ANOVA) tests showed that once the other five predictor’s variables were included only serological test (*p* < 0.01) was significant (Table 2). In multivariable meta-regression the R^2^ was 29%, and the only significant variable was c-ELISA.

**Table 2.** Univariate meta-regression and analysis of variance (ANOVA) results of individual predictors .

S. No	Predictors	Univariate meta-regression^a^		ANOVA^b^	
		Proportion (R^2^) (%)	*p* value (RE)	QM value	*p* value ( RE)
1	Species	2.48	0.09	4.84	0.44
2	Sample size	0.00	0.48	0.09	0.76
3	Serological test	29.20	0.001	47.98	0.001
4	Study region	0.47	0.25	4.15	0.53
5	Study period	2.98	0.07	1.14	0.77

^a^ Proportion of effect of predictors on heterogeneity.

^b^ All variables had a *p* < 0.01 in the fixed effect model.

## 4. Discussion

The systematic review and meta-analysis on seroprevalence of BT showed an overall BT seroprevalence of 39 % in sheep, 43% in goats, 31% in cattle, 35% in buffaloes, 21% in camels, and 66% in Mithun. Few reports showed high susceptibility of domestic and wild ruminants to BTV infection in India (Prasad et al. 1992; Chand et al. 2015). India is one of the important source populations of BTV in Asia along with a few parts of South-East Asia like Indonesia and Malaysia (Rao et al. 2016). Seroprevalence of BTV infection in cattle and buffaloes has been reported from several states of India (Chandel et al. 2004; Prasad et al. 2009). Cattle and goat are sub-clinically infected and act as amplifying hosts in endemic regions. The outbreaks in Indian states are associated with the monsoon period due to peak *Culicoides* activity (Sreenivasulu et al. 2004).

Several serological assays are in use for BT diagnosis. In this analysis, we noted the highest and lowest seroprevalence by c-ELISA (48%, 95% CI: 48 − 49%) and dot-ELISA (6%, 95% CI: 4–7%), respectively. Among the serodiagnostic tests, c-ELISA is reported as highly a sensitive and can be used to detect antibodies raised against all BTV serotypes (Singh and Prasad 2009). Complement fixation test, AGID, and c-ELISA are the OIE recommended methods for BT testing for international trade (Khalid et al. 2012). However, c-ELISA cannot distinguish between infection and vaccination with a live-attenuated vaccine. The serogroup-specific diagnostic tests are AGID, c-ELISA, CFT, indirect or blocking ELISA, fluorescent antibody and dot immunoblotting. Even though AGID was a prescribed test for international trade, it lacks sensitivity, specificity and cross reacts with other Orbivirus serogroups, (Hamblin 2004). The serological response in BTV infection usually appears 7–14 days’ post-infection, and the antibodies can persist between 2 weeks and 6 years after infection (Eschbaumer et al. 2012; Maan et al. 2017). In common, BT seroprevalence in different animals is done either using AGID assay or c-ELISA (OIE 2012). However, dot-ELISA is economical, easy to perform, specific, rapid and could be used for BTV antibody detection at the farm and could be used as an alternative to the c-ELISA. However, the relative superiority of c-ELISA for detection of bluetongue virus antibodies over dot-ELISA is documented (Naresh and Prasad 1995; Ravishankar et al. 2005).

The diagnosis of BTV infection mostly relies on serological methods that identify specific antibodies in serum and AGID is a serogroup-specific test for the detection of BTV antibodies, and was a prescribed test for international trade in earlier years (OIE 2012). Later, c-ELISA was proposed for BT serodiagnosis, which is more sensitive and specific and used to detect antibodies to most of the serotypes and strains of BTV. Conversely, c-ELISA cannot discriminate between the naturally infected animals and BTV vaccinated animals (Maclachlan et al. 2015).

Variations of BT seroprevalence was noticed across regions. This might be due to variations of breed and population density in different regions of India. A previous report correlated BT outbreak in India with climate, sheep population density and breeds (Rao et al. 2016). Ruminants of southern and northern parts of India are susceptible to BT infection where the indigenous sheep breeds show high antibody prevalence (Prasad et al. 2009). Conversely, eastern and north-eastern parts of India are considered to be an un-affected region for BT (Rajkhowa et al. 2008; Joardar et al. 2009). In general, small and marginal farmers of India practice mixed farming where sheep and goats are reared for meat while cattle and buffaloes are maintained primarily for milk production. Though cattle, buffaloes and goats are susceptible to BTV infection, generally they do not show overt clinical signs of the disease (Racloz et al. 2006). Since cattle, buffalo and camels are sub-clinically infected with BTV they may play an important role in BTV transmission and have significant implications in BT control by vaccination in India (Rajkhowa et al. 2008; Rao et al. 2016; Patel et al. 2017). Limited reports on BT in camels show seroprevalence ranging from 21 to 68% (Mozaffari et al. 2013).

All India Network Program on BT (AINP-BT) has been operating in India since 2001, with one of the objectives of surveillance and control. The temporal trend of seroprevalence of BT in sheep, goats, and buffaloes showed an increase from 2001to 2013, suggesting active vaccination during these periods. The decline in BT seroprevalence after 2013, signifies less intensity of vaccination or reduced BT infection. The present study identified regions with high BT seroprevalence and made a base for AINP-BT programme to focus on the high risk regions to reduce the BT infection incidences. Although inactivated pentavalent (BTV serotypes 1, 2, 10, 16 and 23) BT vaccines are available in India (ICAR News Report 2012), vaccination is not regularly practiced in many parts of the country in small ruminants due to limited access of nomadic people keeping sheep and goats. This meta-analysis suggests high heterogeneity among the seroprevalence data between studies and the variations in region level seroprevalence might possibly be due to difference in the agro-climatic conditions, animal population density, and breeds of the animals. During the period between 2002 and 2011 prevalence of antibodies against serotypes BTV-1, BTV-2, BTV-4, BTV-9, BTV-10, BTV-1 2, BTV-16, BTV-21, and BTV-24 have been reported (Rao et al. 2016). According to the current Indian livestock census (19th Livestock Census 2012), India has 65 million sheep, 135 million goats, 191 million cattle, 108 million buffaloes, 0.4 million camels, and 0.29 million Mithun. This meta-analytic study indicates an estimated 25.4 million sheep (95% CI: 21.5–29.9), 58 million goats (95% CI: 51.3–66.2), 67 million cattle, (95% CI: 47.7–86.0), 37 million buffalo (95% CI: 21.7–55.4), 0.06 million camels (95% CI: 0.04–0.09), and 0.19 million Mithun (95% CI: 0.05–0.28) could be BT seropositive in India.

Recently, reverse genetics for BTV has been developed (Boyce et al. 2008), which has boosted BTV research, resulting in various types of next-generation vaccine candidates. New vaccine improvements like modified live vaccines, inactivated vaccines and genetically engineered virus-like particles (VLPs) have been attempted. But all are having certain disadvantages because of nomadic rearing of sheep and goats in India. The lack of a good surveillance system to monitor the circulation of BTV serotypes makes control and eradication of disease in India difficult. Most of the time there is variation in vaccine and field circulating serotypes causing vaccine failure.

Overall, this study provides the pooled estimates of seroprevalence of BT in domestic animals of India by using random-effects model. The study highlights the endemicity of BT in domestic animals of India and difference in seroprevalence across regions. The present study had few limitations. The first one, the important risk factors associated with BT in different animal species of India were not addressed. This could be due to insufficient or lack of reporting the risk factors associated with BT in almost all studies. The second, we used online and offline databases to determine studies on BT seroprevalence, although we did not include unpublished data. The findings of this study signify the need for national and regional seroprevalence surveys to obtain more comprehensive information and identify high-risk areas.

## Supplementary Material

Supplemental Material

Supplemental Material
