# Supplementary material for: Bluetongue in India: a systematic review and meta-analysis with emphasis on diagnosis and seroprevalence
Source: Vet Q. 2020 Sep 23;40(1):229–42. doi: 10.1080/01652176.2020.1810356 (PMC7534259; doi:10.1080/01652176.2020.1810356)
Supplement: Supplemental Material [file TVEQ_A_1810356_SM0975.docx]

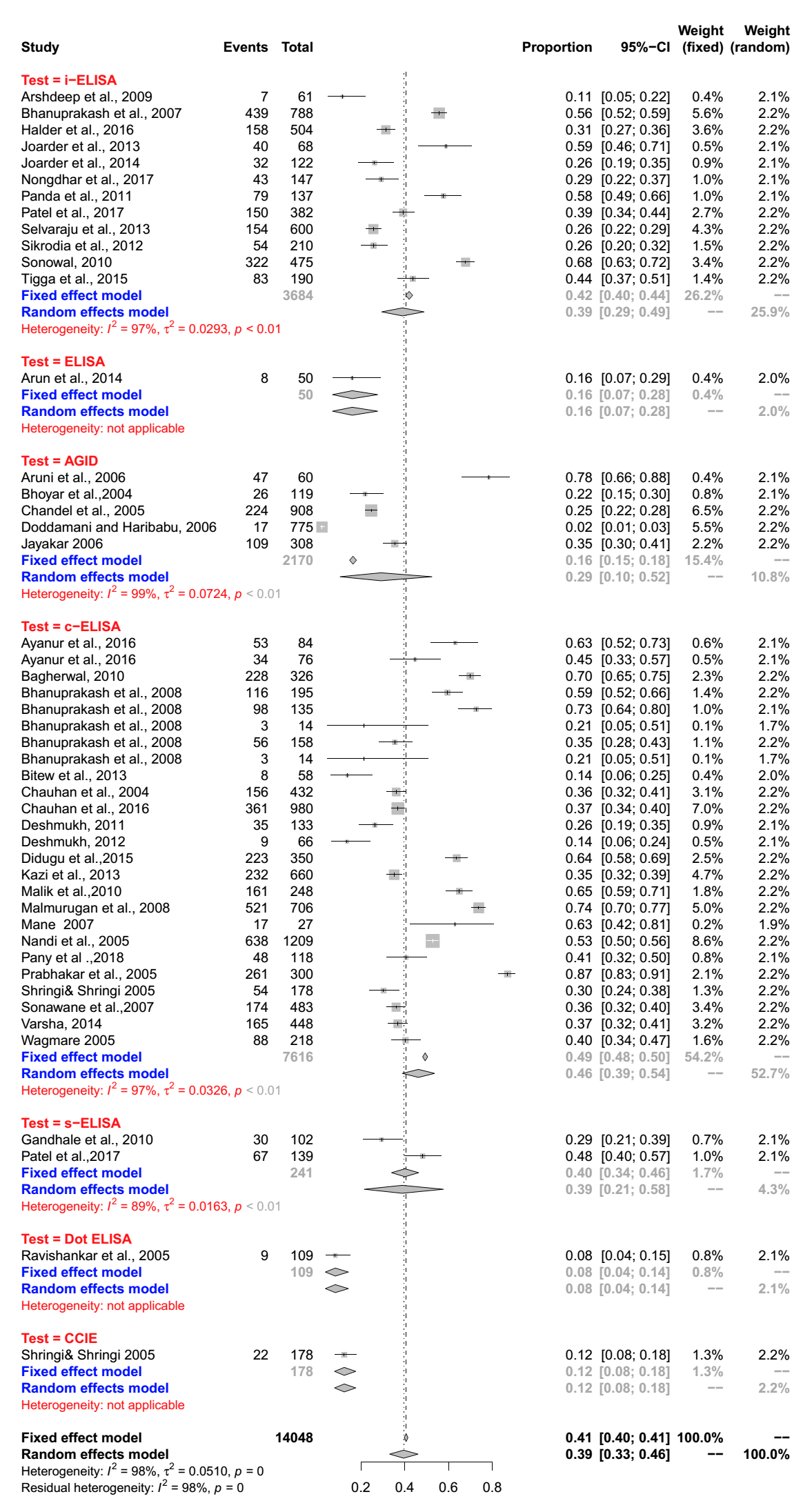


**S. Fig 1: Forest plot showing the diagnostic test wise seroprevalence of BT in sheep**


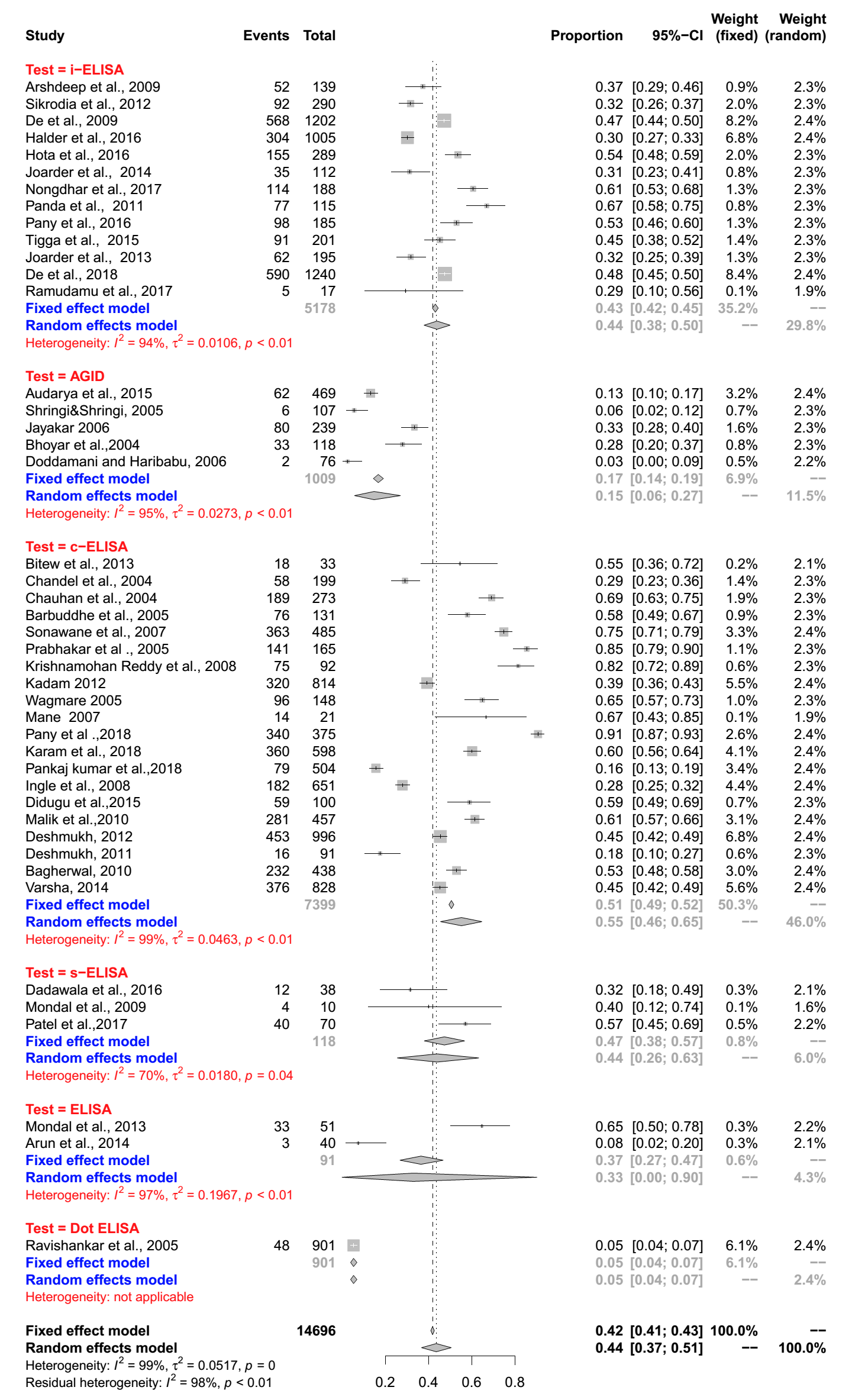


**S. Fig 2: Forest plot showing the diagnostic test wise seroprevalence of BT in goat**


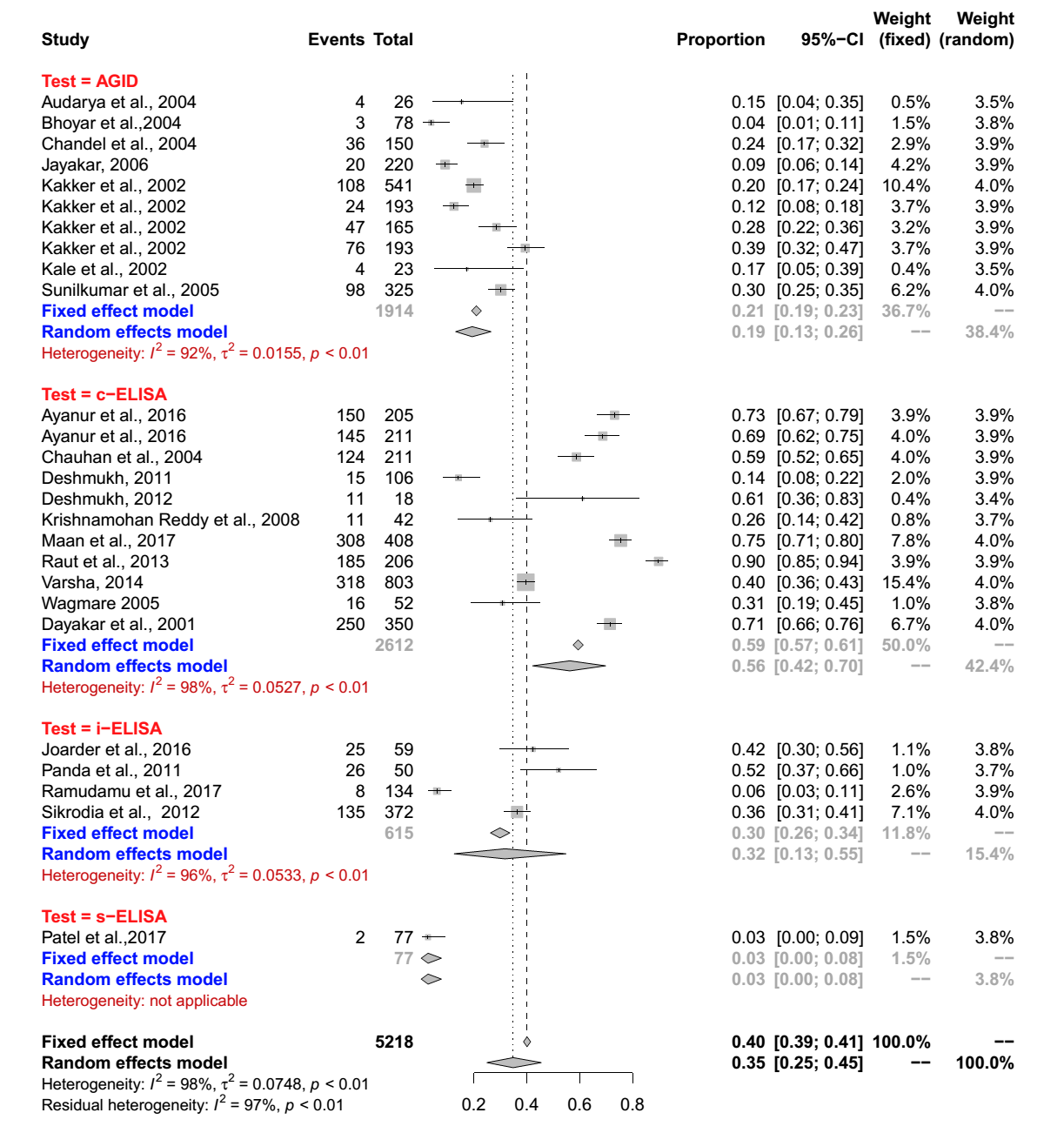


**S. Fig 3: Forest plot showing the diagnostic test wise seroprevalence of BT in cattle**


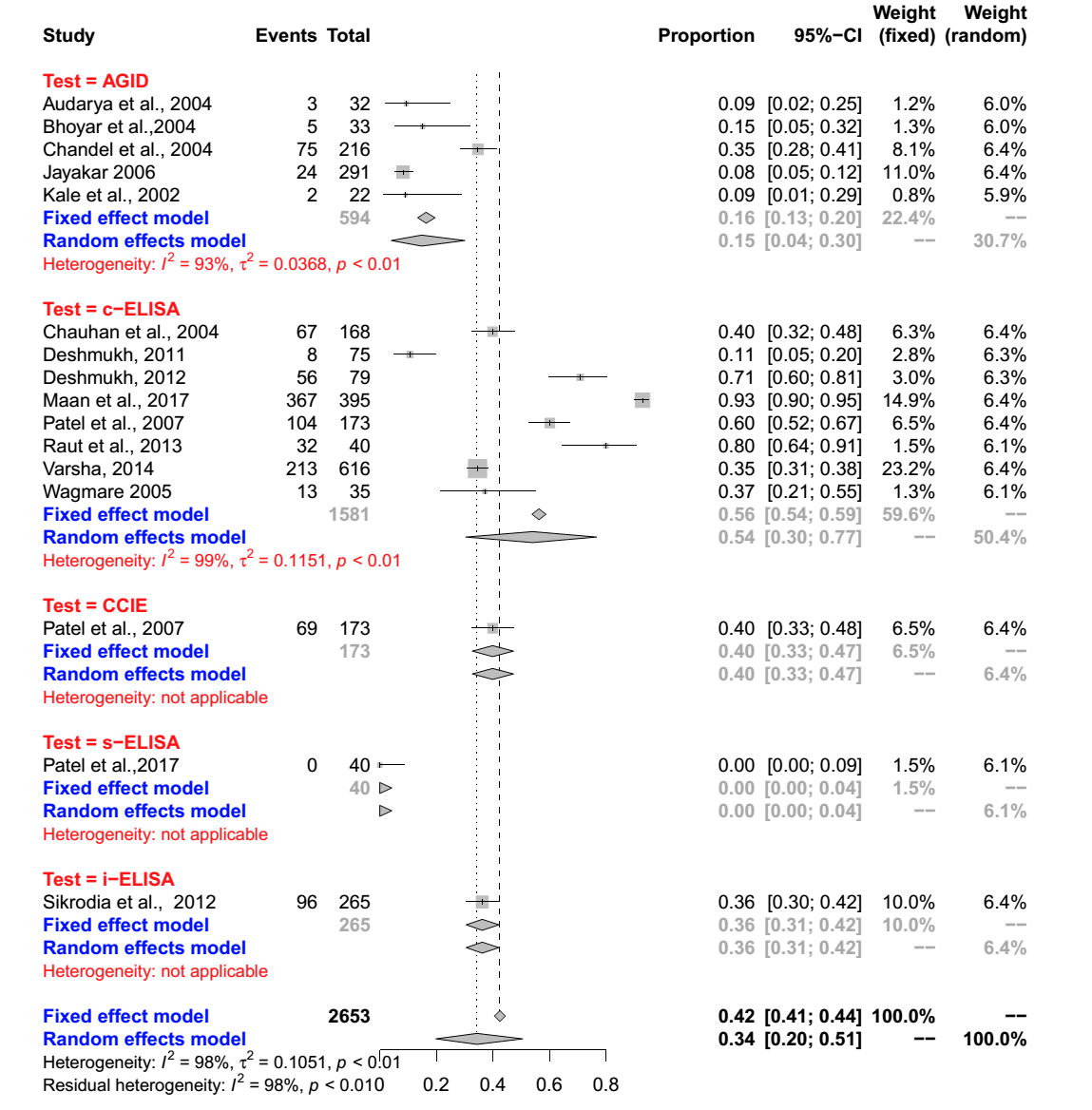


**S. Fig 4: Forest plot showing the diagnostic test wise seroprevalence of BT in buffalo**


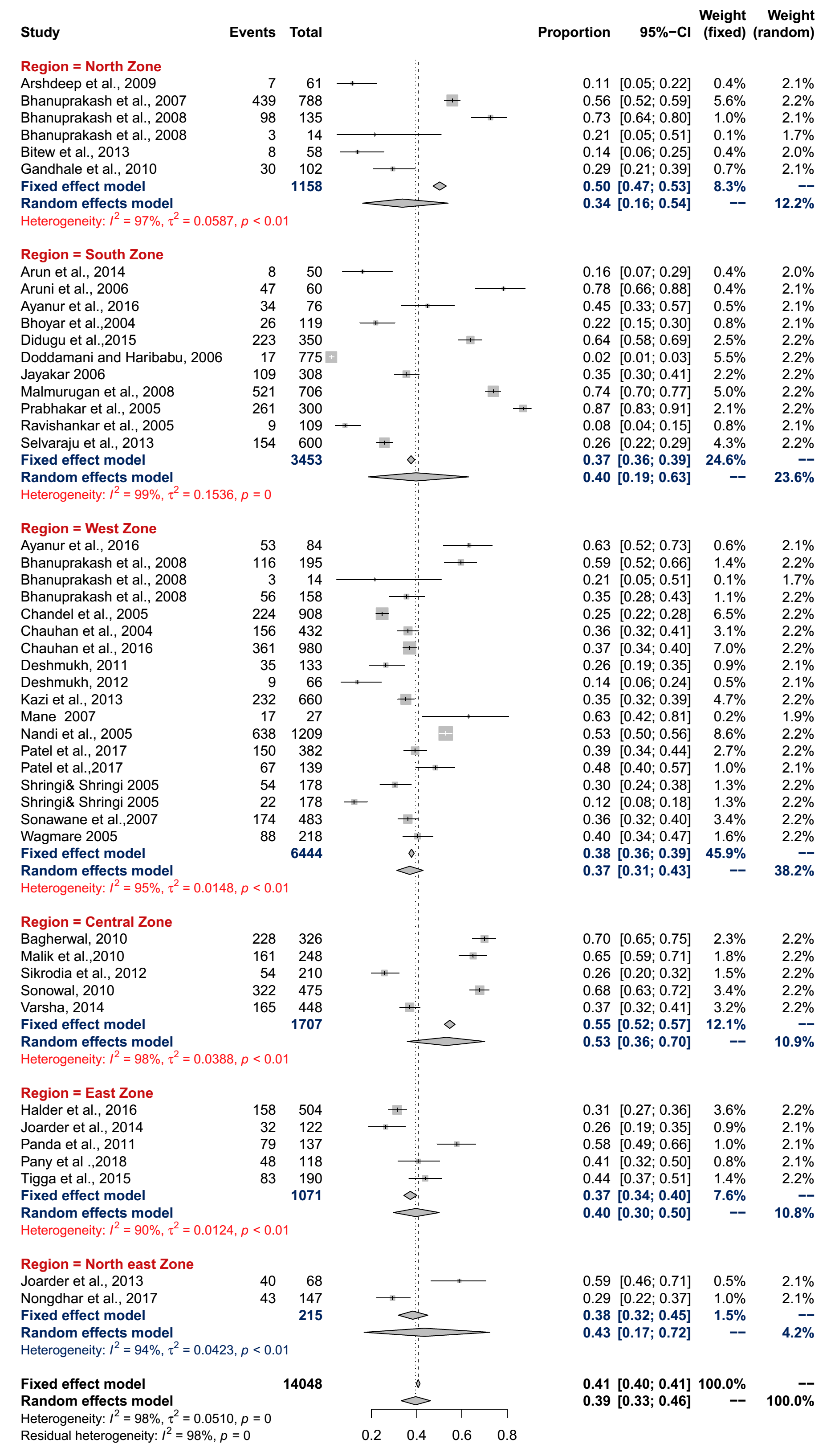


**S. Fig 5: Forest plot showing the region wise seroprevalence of BT in sheep**


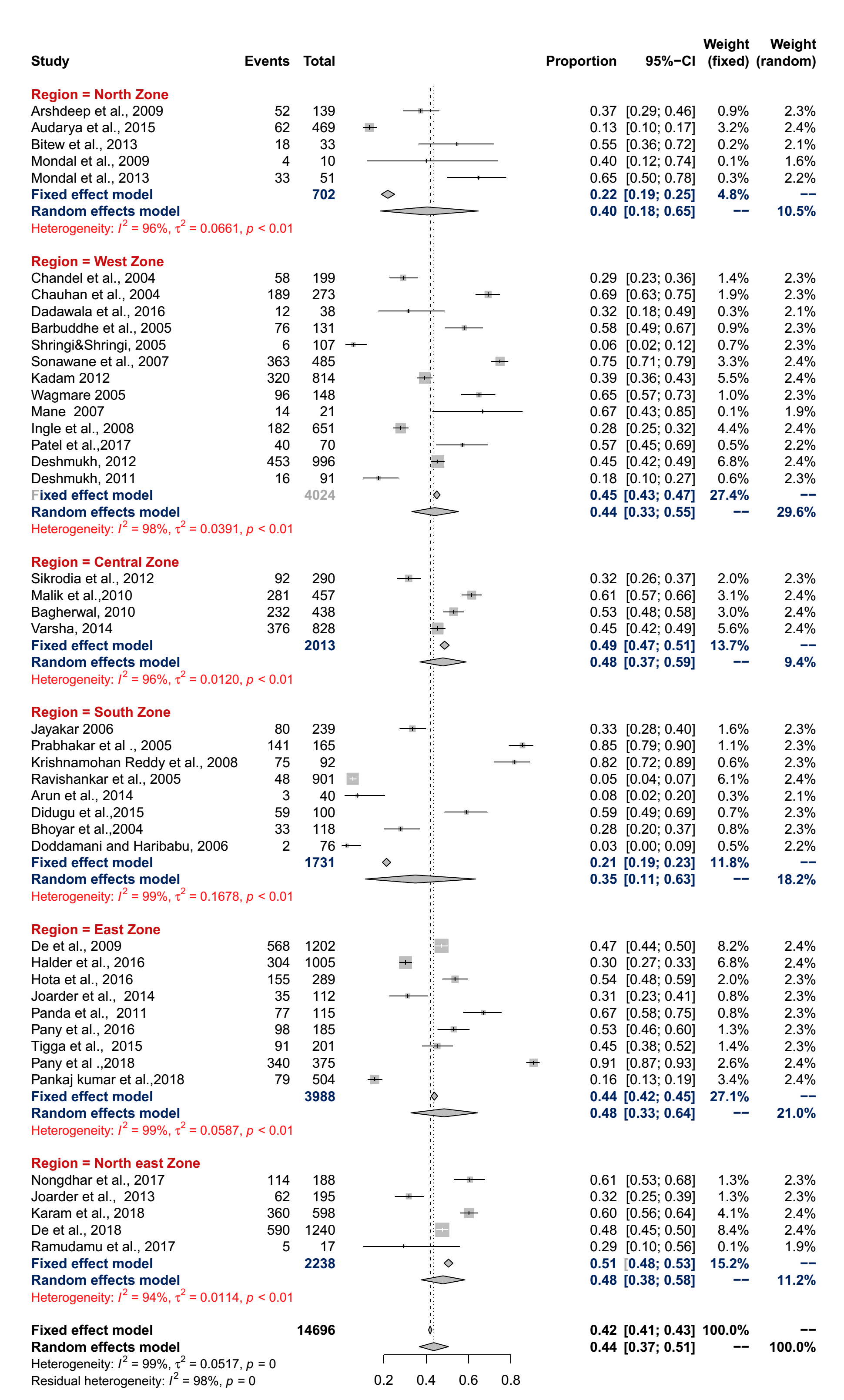


**S. Fig 6: Forest plot showing the region wise seroprevalence of BT in goat**


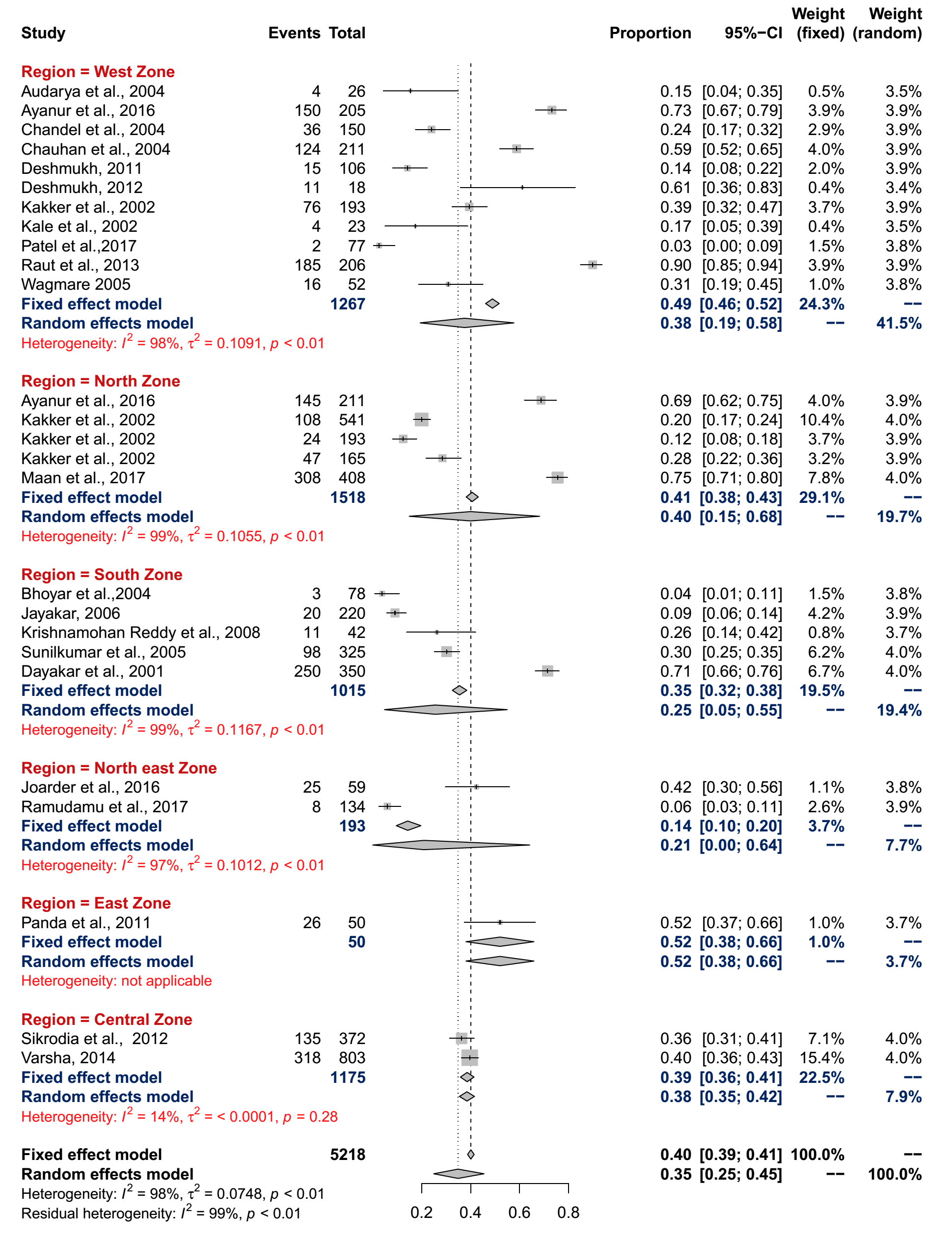


**S.Fig 7: Forest plot showing the region wise seroprevalence of BT in cattle**


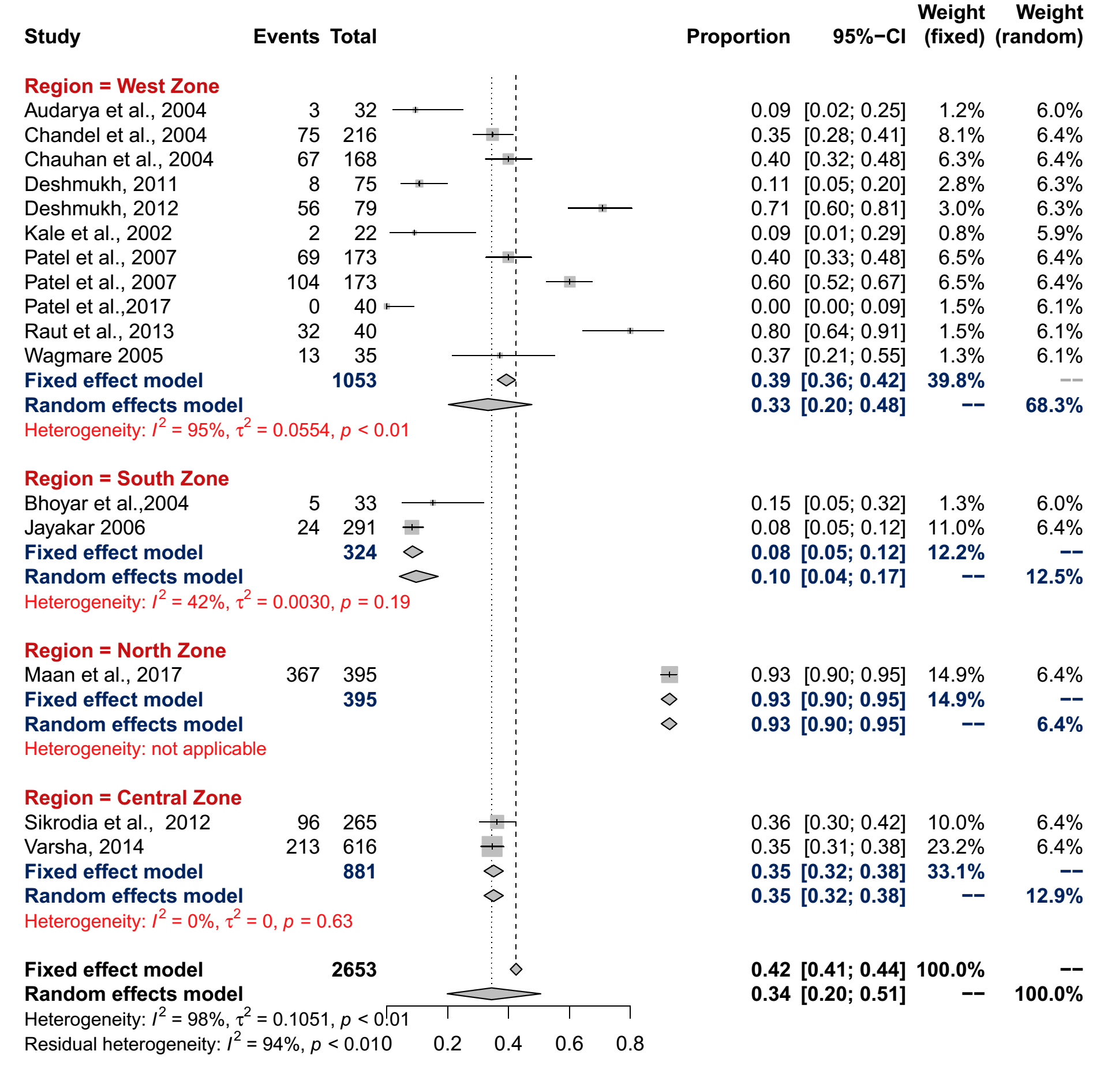


**S. Fig 8: Forest plot showing the region wise seroprevalence of BT in buffalo**


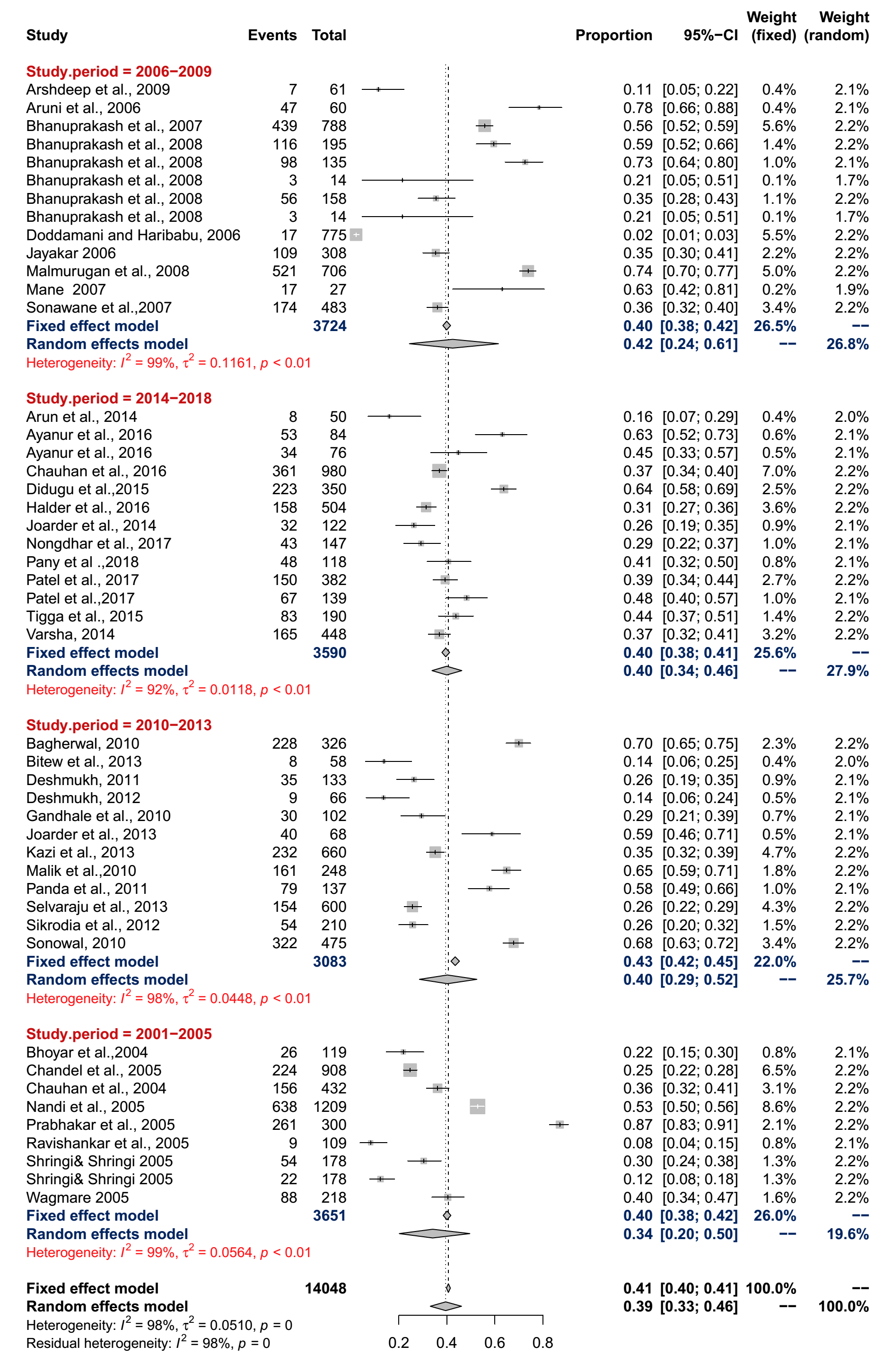


**S. Fig 9: Forest plot showing the study period wise seroprevalence of BT in sheep**


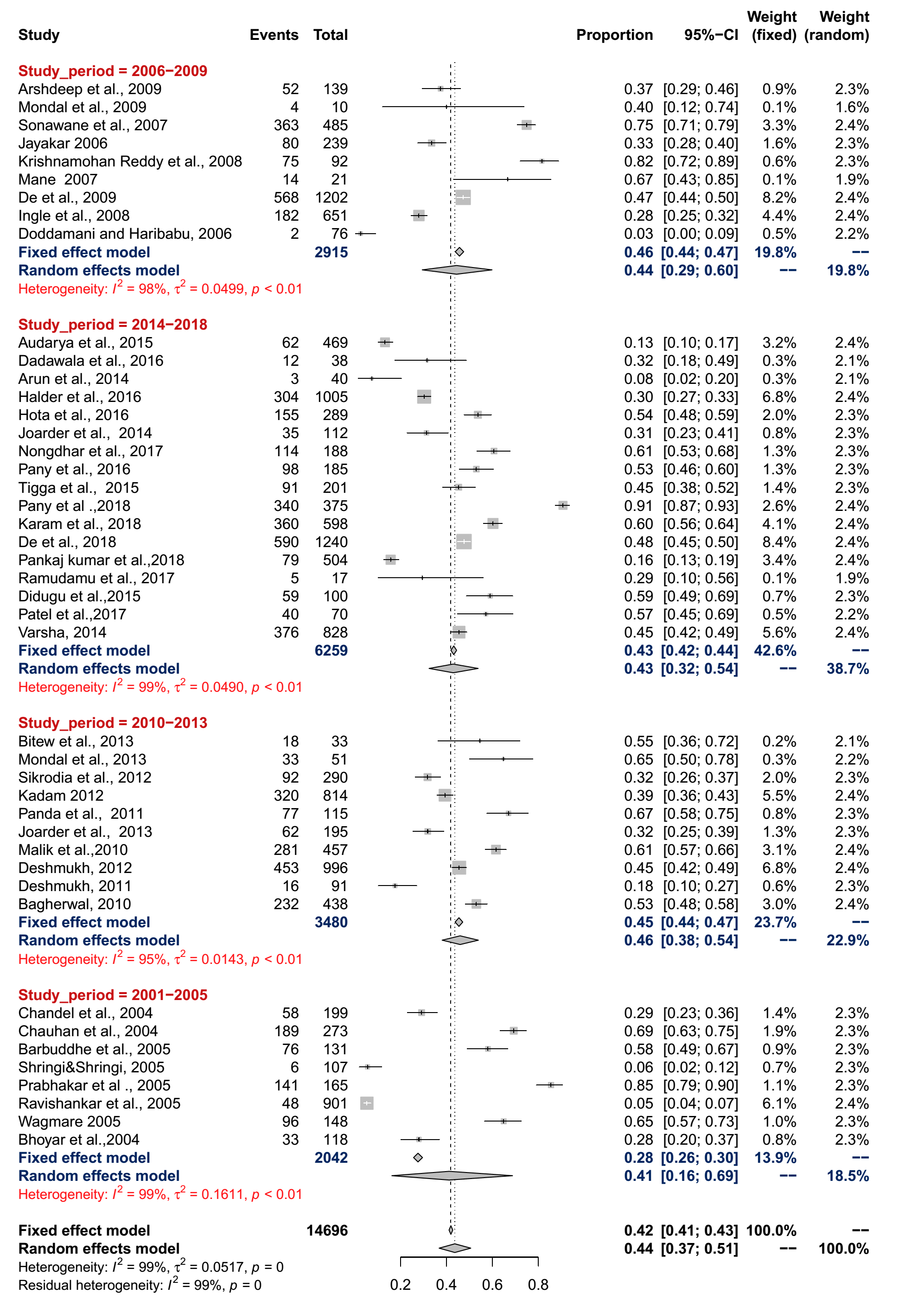


**S. Fig 10: Forest plot showing the study period wise seroprevalence of BT in goat**


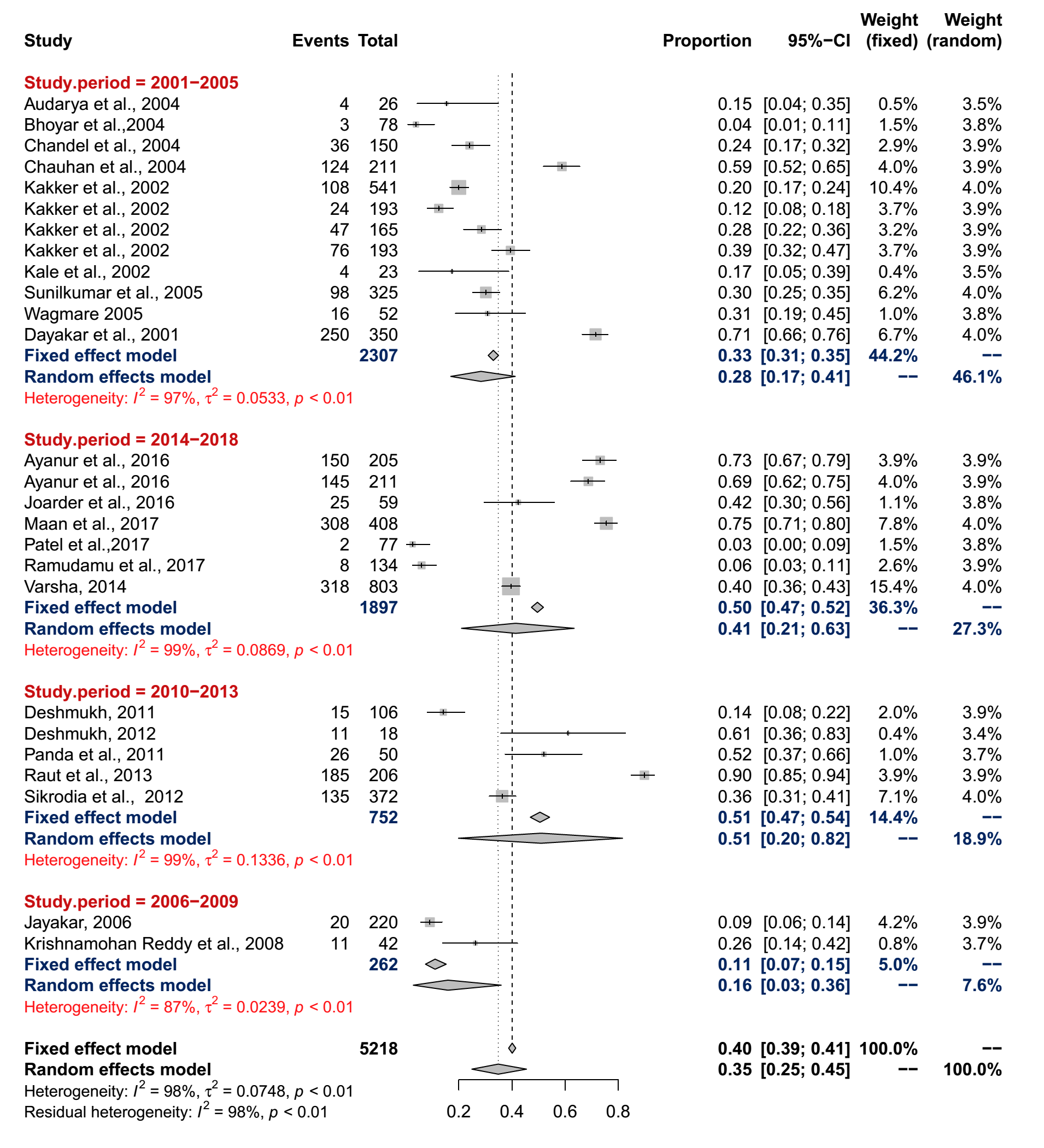


**S. Fig 11: Forest plot showing the study period wise seroprevalence of BT in cattle**


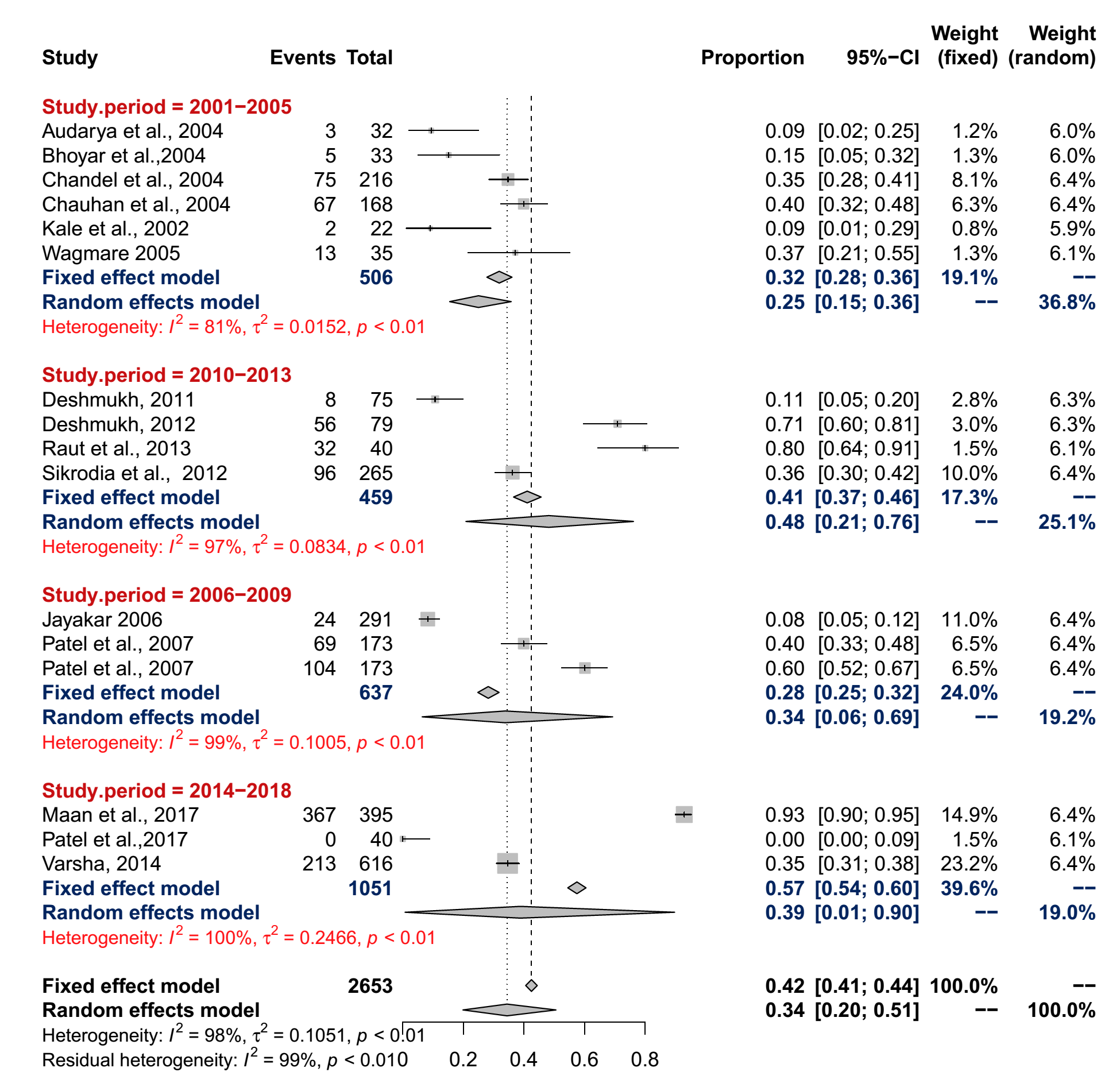


**S. Fig 12: Forest plot showing the study period wise seroprevalence of BT in buffalo**


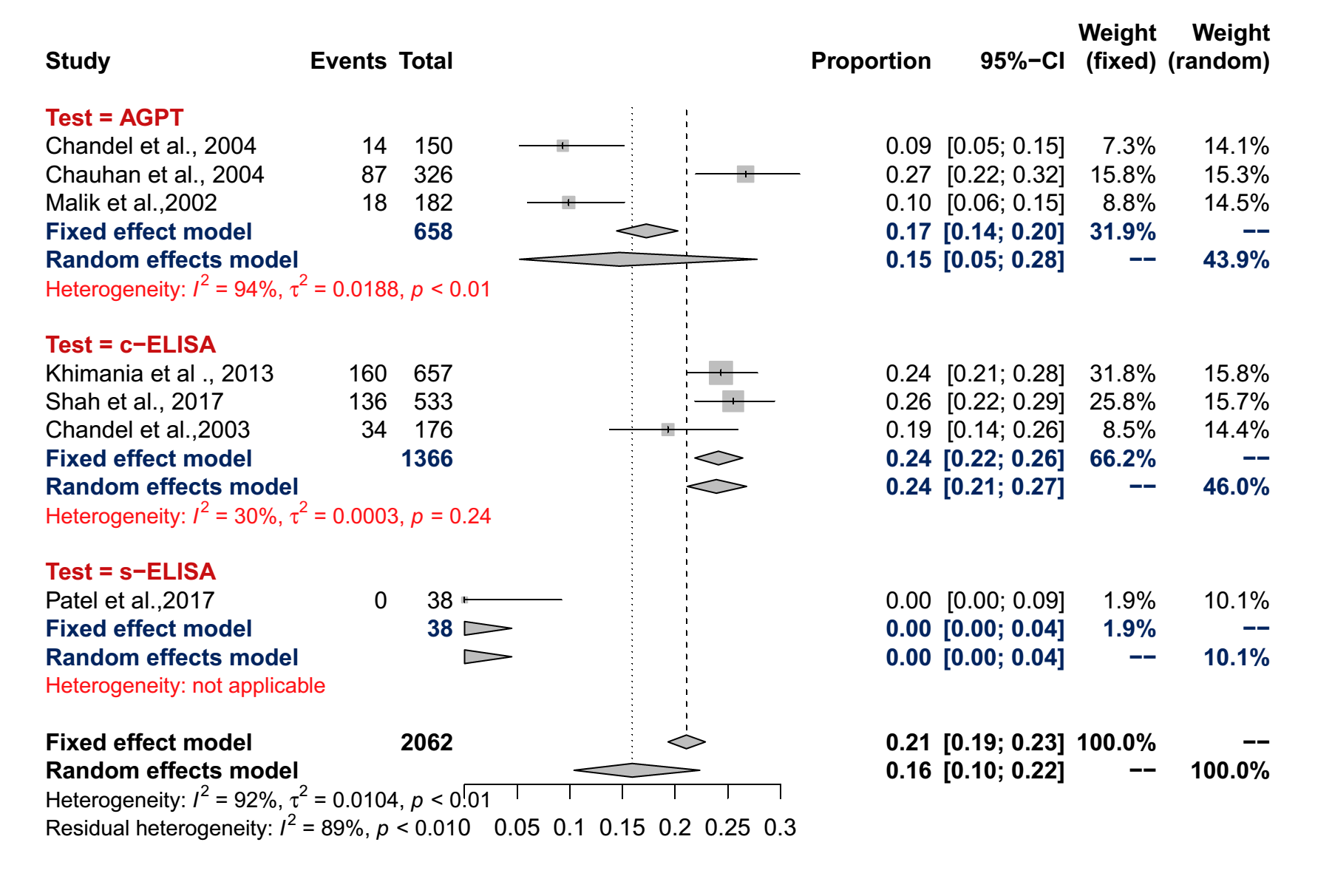


**S. Fig 13: Forest plot showing the diagnostic test wise seroprevalence of BT in Camel**


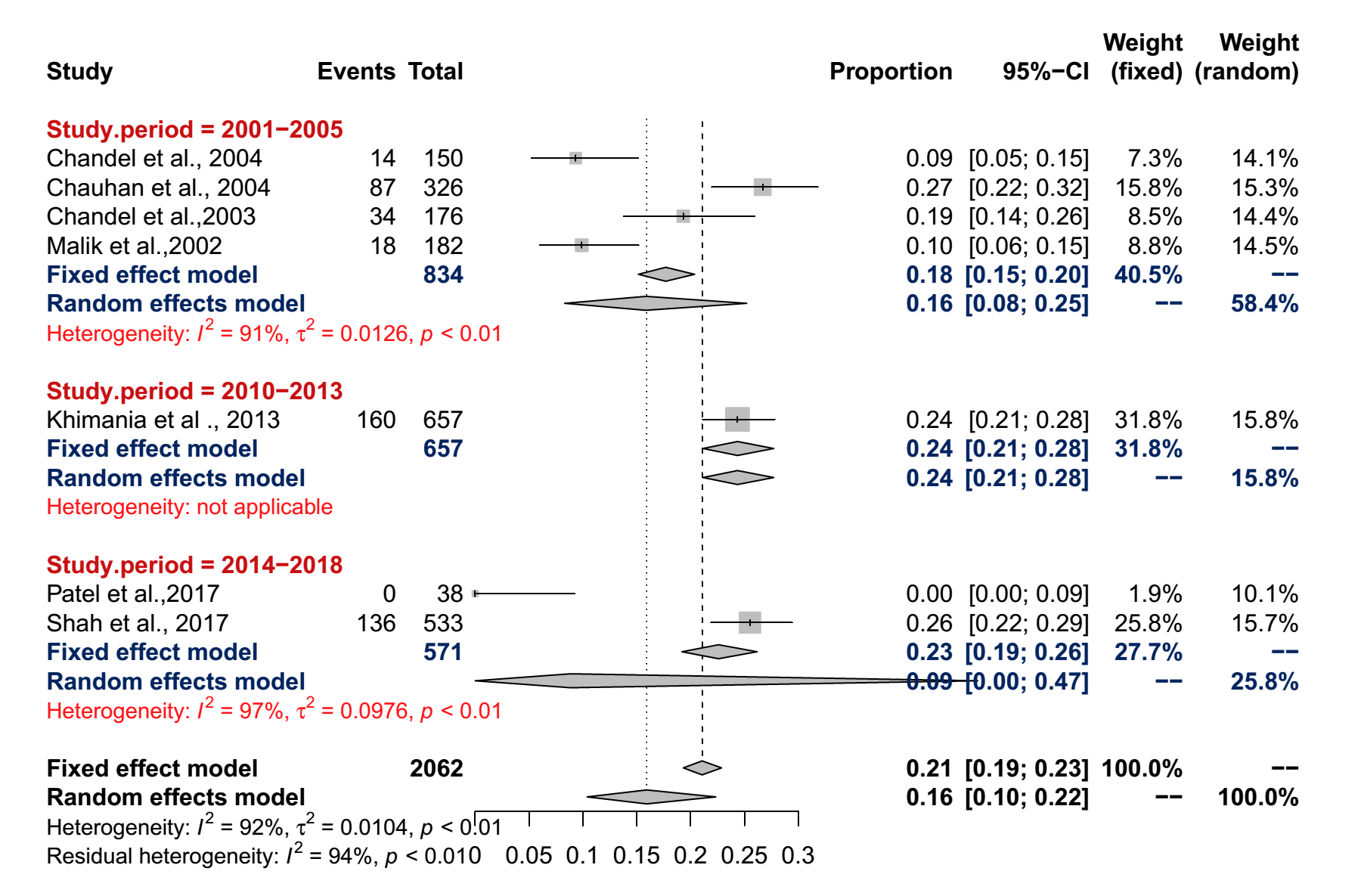


**S. Fig 14: Forest plot showing the study period wise seroprevalence of BT in Camel**
